# High-Throughput Testing for Unknown Mutagens and Cytotoxica via Duplex Planar Ames–Cytotoxicity Bioassay Including Metabolic S9 Activation

**DOI:** 10.1021/acs.analchem.5c06690

**Published:** 2026-03-02

**Authors:** Katharina Schmidtmann, Ann-Cathrin Kayser, Gertrud E. Morlock

**Affiliations:** Chair of Food Science, Institute of Nutritional Science, 98899 Justus Liebig University Giessen, Heinrich-Buff-Ring 26-32, Giessen 35392, Germany

## Abstract

Current nontarget effect-directed analysis of complex samples for mutagens is hampered by matrix effects, associated cytotoxicity, diffusion effects, insufficient sensitivity, and a lack of selectivity. Non-target analysis may overlook highly potent, unknown mutagens at trace levels. To overcome these limitations, a duplex planar Ames mutagenicity–cytotoxicity bioassay was developed to sensitively and selectively detect individual mutagens and cytotoxic compounds with or without metabolic activation. Key innovations included high-throughput testing of samples, either directly or as raw extracts, separated in parallel by planar chromatography, substance zone fixation to prevent diffusion during long incubation times, integration of the human versus rat liver S9 enzyme systems for metabolic de/activation, and use of a tetrazolium salt substrate that provides a dual end-point read-out. Compared to the state of the art, time to result was reduced 5-fold, manual work was reduced 330-fold, and costs for nearly plastic-free consumables were reduced 651-fold. The selective, sensitive, and quantitative unmasking of previously unknown mutagens and cytotoxica was shown for highly complex samples, such as teas, cosmetics, skin care creams, and perfumes. An exemplary daily exposure to 11.5 g of skin care products exceeded the half-maximal effective mutagenicity dose by at least 4 orders of magnitude. Using the open-source and sustainable 2LabsToGo-Eco, the new duplex planar bioassay method can be applied worldwide to serve as a valuable tool for hazard minimization and to support regulatory safety and risk assessments, industrial quality control, and drug development.

## Graphical abstract

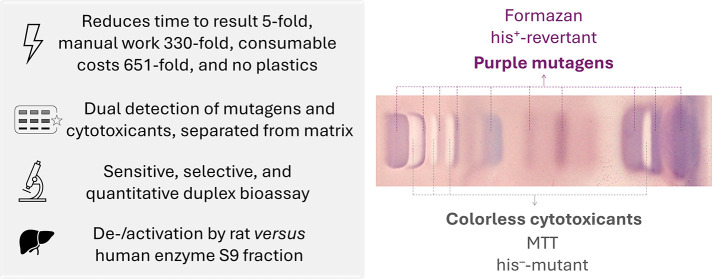

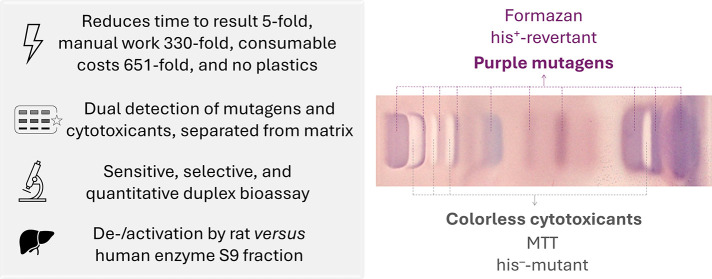

## Introduction

Nontarget analysis of complex samples for mutagens faces significant limitations. Concentration-based methods, such as high-performance liquid chromatography–high-resolution mass spectrometry (HPLC–HRMS/MS), overlook unknown mutagens and highly potent mutagens at the ultratrace level.^1^ Effect-based methods, such as *in vitro* microtiter plate mutagenicity bioassays (which provide only a sum value), can hinder or mask the detection of mutagens due to insolubility, solidification, precipitation, micelle formation, adsorption to plastic surfaces, associated cytotoxicity, false positives, false negatives, *etc*.^1^ Among the mutagenicity bioassays, the Ames assay is the oldest^2–5^ and the most widely used over the past five decades, due to its cost-effective workflow that requires only standard laboratory equipment. Although taking 3 days, it is considered more simple and robust than other mutagenicity assays (*e.g*., effortful mammalian cell tests are prone to false-positive results due to variations in parameters such as pH and osmolarity effects) and has a mutagenicity predictivity of approximately 70–90% when compared to at least one positive result in one of the following *in vitro* mammalian cell tests.^6^ For the Ames assay, different *Salmonella* Typhimurium strains can be used, including TA1535, TA1537, TA97, TA97a, TA98, and TA100.^7^ However, the strains TA98 and TA100 alone are sufficient to detect 93% of known mutagens, as reported in 2019,^8^ which was confirmed to be 94% in 2023.^9^ Several modified *in vitro* formats have been developed, including the Ames microtiter plate format (MPF) assay^10,11^ and a microtiter plate Ames assay with luminescent reporter strains.^12^ However, matrix interferences compromised the detection of mutagenic compounds when the Ames MPF assay was used for screening complex samples, such as food contact materials.^13^

The *in vitro* microtiter plate assay analysis of complex samples is rarely reported, as the sum value is prone to errors. Complex matrices, such as teas, cosmetics, skin care creams, and perfumes, severely impair mutagen analysis, making accurate results impossible. Tea is regarded as a health-promoting beverage^14^ due to its high polyphenol content, including catechins, theaflavins, and flavonoids.^15–17^ While black and especially green teas are widely recognized for their antimutagenic and anticancer properties,^18–20^ primarily attributed to their high polyphenol content,^18,19^ the same complex mixtures could also mask the detection of mutagens.^21,22^ Less attention has been paid to the possibility that certain compounds among the many thousands of different tea compounds may exhibit mutagenicity. As a further example, cosmetics and skin care creams contain a wide range of value-adding or even functional ingredients, either natural or synthetic, often mineral-oil-based, and numerous synthetic excipients, which increase shelf life or form the physical matrix for functional release and technical processability. Recent studies have shown that functional ingredients, such as retinyl palmitate, can undergo photodegradation to mutagenic products upon UVA exposure, highlighting a potential genotoxic risk inherent to certain cosmetic additives.^23^ Some UV filters added for light stability of the products have been found to induce DNA damage *in vitro*.^24,25^ Further, the impurities ethylene or diethylene glycol of widely used synthetic excipients can be metabolized in the body to the mutagenic glycolaldehyde, glyoxal, and glyoxylic acid.^26–28^ Natural lipophilic components of cosmetic formulations (such as waxes, oils, or fats) can be oxidized or contaminated at the trace level with highly potent mutagenic peroxides or epoxides of polycyclic aromatic hydrocarbons (PAHs),^29,30^ mineral oil saturated/aromatic hydrocarbons (MOSH/MOAH),^31,32^ and chlorinated paraffins.^33^ Unfortunately, more or less refined mineral oil fractions^34^ are the main ingredient of mineral-oil-based creams.^35^ As the last example, perfumes are an ethanol-based mixture of fragrances composed of thousands of natural or synthetic compounds based primarily on mineral oil.^36,37^ Recently, the chemical safety of perfumes,^38–40^ as well as cosmetics and skin care creams,^41^ has been questioned based on the many hazardous compounds detected therein via planar bioassay screening.

Combining mutagenicity testing with prior chromatographic separation could be a game-changer in handling complex samples. An agar-overlay bioautography for mutagenicity testing on a thin-layer chromatography plate, and subsequent cell counting, was already reported in 1982.^42^ The detection limit for a typical mutagen, such as 4-nitroquinoline-*N*-oxide (4NQO), was about 1 μg per zone. However, disadvantages included short incubation times to prevent excessive diffusion, questionable attribution of colonies to specific zones, and poor reproducibility due to manual agar application and variable agar layer thickness.^42,43^ Recently, the Ames MPF *in vitro* assay format^10,11,44^ was transferred to a planar bioassay format on high-performance thin-layer chromatography (HPTLC) plates.^45^ However, the results were poor, given the short incubation time (limited to 5 h due to zone diffusion) and detection via a nonselective substrate. Both *in vitro* and on-surface assay formats utilize bromocresol purple as a pH indicator substrate, which detects acidification resulting from metabolic activity as an indirect proxy for bacterial growth by exhibiting a distinct color change from purple to yellow. However, bromocresol purple showed low selectivity for mutagens, as it also responded to acidic compounds such as plant acids, organic acids, sugar acids, and acidic metabolization products.

To improve detection sensitivity and selectivity, as well as zone resolution,^45^ this study aimed to design a new, more powerful planar Ames bioassay. A reliable mutagenicity screening, including quantitative results such as the half-maximal effective mutagenicity dose (ED_50_), should be demonstrated for highly complex samples in daily use, such as teas,^45^ cosmetics and skin care creams,^41^ and perfumes.^38^ It was intended to compare the predominant metabolization via the rat liver S9 enzyme system^46,47^ with a new human liver S9 enzyme system.^48^ Any coherence between the new mutagenicity and recent genotoxicity testing results of the planar SOS-Umu-C bioassay screening^49,50^ should be figured out. The method should be transferred to the affordable open-source 2LabsToGo-Eco^51^ to outline the full potential and progress achieved in the field of effect-directed analysis (EDA).

## Experimental Section

### Chemicals and Materials

Ethyl acetate (>99.8%) and dichloromethane (>99.9%) were procured from Th. Geyer (Renningen, Germany). Methanol was delivered by VWR International (Darmstadt, Germany). 4-Nitroquinoline-*N*-oxide (4NQO, >98%) was sourced from Tokyo Chemical Industry (Tokyo, Japan). N4-Aminocytidine (N4ACT, 95%) was obtained from Angene Chemical (London, England). 2-Acetylaminofluorene (2AAF, > 98%), 2-aminofluorene (2AF, > 98%), and *tert*-butylbenzene (TBB, >98%) as MOAH candidates were from TCI (Eschborn, Germany). Glycerol (>99%), magnesium sulfate heptahydrate (≥99%), ammonia (25%), 2-aminoanthracene (2AA, 96%), primuline (50%), sodium ammonium hydrogen phosphate tetrahydrate (≥99%), resorufin-β-D-galactopyranoside (95%), lauric acid (La, ≥98%), linoleic acid (L, 60–74%), triolein (O_3_, ≥ 99%) as a triacylglycerol (TAG) candidate, perylene (>98%) as a MOAH candidate, and bicyclohexyl (>99%) as a MOSH candidate were purchased from Fluka Sigma–Aldrich (Steinheim, Germany). Ampicillin sodium salt (>99%), thiazolyl blue tetrazolium bromide (MTT, 3-(4,5-dimethylthiazol-2-yl)-2,5-diphenyltetrazolium bromide, >98%), acridine orange (AO, ≥99%), disodium hydrogen phosphate (99%), potassium chloride (≥99%), citric acid monohydrate (≥99.5%), potassium dihydrogen phosphate (≥99%), dimethyl sulfoxide (≥99.8%), magnesium chloride (MgCl_2_, ≥99%), glucose 6-phosphate (G-6-P, ≥98%), dipotassium hydrogen phosphate (≥99%), and capric acid (Ci, ≥98%) were obtained from Carl Roth (Karlsruhe, Germany). Cyclohexane (99.8%), nutrient broth No. 2 medium, and 5-α-cholestane (>99%) as a MOSH candidate were provided by Thermo Fisher Scientific (Darmstadt, Germany). α-Linolenic acid (αLn, 99%) and 2-methylnaphthalene (2MN, >96%) as MOAH candidates were purchased from Acros Organics (Geel, Belgium). Monoolein (O_1_, ≥99%) as a monoacylglycerol (MAG) candidate, diolein (O_2_, > 99%) as a diacylglycerol (DAG) candidate, tristearin (>99%, S_3_) as a further TAG candidate, 3-monochloropropane-1,2-diol (MCPD, ≥99.5%), and 3-monochloropropane-1,2-diol fatty acid esters (MCPDE, ≥99%) were obtained from Larodan (Solna, Sweden). Tridecane (C13, >98%) as a MOSH candidate was purchased from Biozol (Hamburg, Germany). Stearic acid glycidyl ester (SGE, ≥95%) was procured from Cayman Chemical (Hamburg, Germany). A mixture solution of polychlorinated alkanes (long-chain chlorinated paraffins CP18–20, 49% Cl, 100 ng/μL in cyclohexane) was purchased from Dr. Ehrenstorfer (Augsburg, Germany). Cryopreserved cell pellets of *Salmonella* Typhimurium (serovar, mutant) strains TA98 and TA100 were purchased from Trinova Biochem (Giessen, Germany). The HiCultS9 (cultivated, cell line-derived human enzyme S9 fraction) was delivered by Scinora (Rafz, Switzerland), and the nicotinamide adenine dinucleotide phosphate (NADP, 99%) was obtained by BLDpharm (Shanghai, China). The rat liver S9 system was purchased from Xenometrics (Allschwil, Schweiz) and consisted of ready-to-use solutions of buffer salts, S9 fraction, NADP, buffer M, and G-6-P. High-performance thin-layer chromatography (HPTLC) plates silica gel 60 (20 × 10 cm) and sodium chloride (≥99%) were supplied by Merck (Darmstadt, Germany). Degalan P28 N (99%, polyisobutyl methacrylate polymer) was donated by Röhm (Darmstadt, Germany). Double-distilled water was produced using a Heraeus Destamat B-18E system (Thermo Fisher Scientific). The 35 samples (5 tea drugs, 22 cosmetics and skin care creams, and 8 perfumes; Table S1) were purchased from local supermarkets or drugstores. Product names and manufacturers were not published to protect the companies.

### Preparation of Media, Substrate, HiCultS9 Cofactor, Degalan, and Positive Control Solutions

For the growth medium (ISO 11350 ^52^), 12.5 g of nutrient broth No. 2 medium (special meat extract containing histidine 10.0 g/L, peptone 10.0 g/L, and sodium chloride 5.0 g/L) was dissolved in 0.5 L of double-distilled water (pH 6.5) and autoclaved. For the deficiency medium (ISO 11350 ^52^), 0.2 g of magnesium sulfate heptahydrate, 2 g of citric acid, 10 g of dipotassium hydrogen phosphate, and 3.5 g of sodium ammonium hydrogen phosphate tetrahydrate were dissolved in 0.5 L of double-distilled water (pH 6.5) and autoclaved. The MTT substrate solution (2 mg/mL) was prepared in phosphate-buffered saline (PBS) buffer (0.1 g of potassium dihydrogen phosphate, 0.7 g of disodium hydrogen phosphate, 4.0 g of sodium chloride, and 0.1 g of potassium chloride dissolved in 0.5 L of double-distilled water). For HiCultS9 cofactor solutions, the NADP (300 mM), G-6-P (1.5 M), and MgCl_2_ (500 mM) were prepared in double-distilled water. For zone fixation, 2 mL of 0.5% Degalan in acetone was mixed with 0.66 mL of ethanol. The media were stored in the refrigerator for up to 4 months, while the buffer solutions and cofactors were stored for up to 8 months. For positive control solutions, N4ACT was dissolved in PBS buffer (0.5 mg/mL), whereas 4NQO, 2AA, 2AF, and AO were dissolved in methanol (1 mg/mL each). The reference substances (Ci, La, L, αLn, O_1_, O_3_, S_3_, 10 mg/mL each), O_2_ (2 mg/mL), SGE (5 mg/mL), MCPD (100 mg/mL), and MCPDE (100 mg/mL) were dissolved in *n*-hexane. For the MOSH/MOAH standard, C13 (300 ng/μL), bicyclohexyl (300 ng/μL), 5-α-cholestane (600 ng/μL), perylene (50 ng/μL), 2MN (600 ng/μL), and TBB (600 ng/μL) were altogether dissolved in toluene. All solutions were stored in the refrigerator for up to 4 months, except for the positive control and reference solutions, which were stored for up to 12 months.

### Preparation of Cryostocks from Overnight Cultures of TA98 and TA100

Cryopreserved cell pellets of the two *S.* Typhimurium strains TA100 and TA98 were cultured, each in 35 mL of growth medium at 37 °C (incubator Cultura M 70700R, Almedica, Galmiz, Switzerland) with constant shaking (120 rpm, orbital shaker, Bühler, Bodelshausen, Germany) for 16 h. Cryostocks were prepared by centrifuging a 20 mL aliquot of the cell suspension at 3,000 × g (Centrifuge 5702, Eppendorf, Hamburg, Germany) for 10 min. The supernatant was discarded. The resulting cell pellet was resuspended in 20 mL of precooled growth medium containing 10% glycerol, then transferred and aliquoted into 10 2 mL cryovials and stored at −80 °C. For overnight cultures, 10 μL of each strain (cryostock) was inoculated into 35 mL of growth medium containing 35 μL of ampicillin solution (100 mg/mL in double-distilled water) and incubated at 37 °C at 120 rpm for 14–16 h.

### Positive Control Band Pattern Test

Bands (8 mm) of increasing volumes (amounts) of the positive control solutions (4NQO 0.1–2 μg/band, N4ACT 1–20 μg/band, and DMSO 100–1000 μg/band; with S9 metabolization, 2AF, AO, 2AA each 0.001–10 μg/band) were applied as a band pattern (above each other) with the following settings: 10 or 25 μL syringe volume, 150 nL/s dosage speed, 200 nL predosage volume, 15 μL/s filling speed, 4 s vacuum time, and 4 s rinsing time (Automatic TLC Sampler ATS 4, CAMAG, Muttenz, Switzerland, operated via visionCATS v3.2 and winCATS v1.4.7). The band pattern was dried with a cold stream of air (a hair dryer, unless otherwise stated) for 5 min.

### Preparation and HPTLC Separation of Three Different Sample Types

Liquid samples were used directly, whereas solid samples required extraction.

- Each tea drug was extracted with methanol (100 mg/mL). After ultrasonification (75 °C, 30 min, Sonorex Digiplus, Bandelin, Berlin, Germany) and centrifugation (17,000 × g, 15 min, Eppendorf 5702 centrifuge, Heraeus, Hanau, Germany), each supernatant was filtered (0.22 μm pore size, membrane diameter 33 mm, cellulose mixed esters (CME) syringe filter, Carl Roth, Karlsruhe, Germany) into a sampler vial. For analysis, 30 μL (3 mg) of each extract was applied as a 3 × 8 mm^2^ area onto an HPTLC plate silica gel 60 as mentioned, but with a 200 nL/s dosage speed (ATS 4), followed by drying (2 min) and development with 7 mL of dichloromethane–methanol–ammonia 85:15:1, *V*/*V*/*V*, up to 70 mm (Twin-Trough Chamber, CAMAG).^45^
- Each cosmetic or skin care cream was extracted with a mixture of double-distilled water, methanol, and ethyl acetate 0.2:1:1, *V*/*V*/*V* (100 mg/mL), ultrasonicated (25 °C, 10 min), centrifuged (17,000 × g, 10 min), and transferred to a sampler vial. For analysis, 8 μL (0.8 mg) of each extract (or upper phase for liquid cosmetics) was applied as an 8 mm band onto an HPTLC plate silica gel 60 as mentioned, but with a 200 nL/s dosage speed (ATS 4), followed by drying (2 min), chamber saturation with 14 mL mobile phase for 5 min, and development with 10 mL pentane–diethyl ether 8:3, *V*/*V*, up to 75 mm.^41^
- Each perfume was directly transferred into a sampler vial, applied as an 8 mm band (1 μL) onto an HPTLC plate silica gel 60 as mentioned, followed by drying (2 min) and development with 7 mL of cyclohexane–ethyl acetate 19:1, *V*/*V*, up to 70 mm.^38^

Each plate was dried (5 min) and detected under white light illumination (Vis) and fluorescence detection (FLD) at 254 and 366 nm (Visualizer 2, CAMAG). All extract solutions were stored tightly closed in the refrigerator for up to 4 months.

### Duplex Planar Ames–Cytotoxicity Bioassay–Vis

The positive controls 4NQO (1 μg/band), DMSO (1000 μg/band), or N4ACT (0.6 μg/band) were applied above the solvent front of the chromatogram and dried (1 min). For zone fixation, 2 mL of Degalan solution was piezoelectrically sprayed (green nozzle, level 4, Derivatizer, CAMAG) onto the chromatogram and dried (5 min). Each overnight culture was adjusted to an optical density at 600 nm (OD_600_) of 0.4 with the growth medium (Spectronic CamSpec, West Yorkshire, UK). Each cell suspension (2.5 mL) was pipetted into a 15 mL centrifugation tube (Sarstedt, Nümbrecht, Germany) and centrifuged at 3,000 × *g* for 10 min. Each supernatant was discarded. The deficiency medium (2.5 mL) was used for cell resuspension, and the entire cell suspension was piezoelectrically sprayed (red nozzle, level 4, Derivatizer) onto the HPTLC chromatogram. The seeded plate was placed in a polypropylene box (KIS 26.5 cm × 16 cm × 19 cm, ABM, Wolframs–Eschenbach, Germany); lined with filter paper premoistened with 40 mL of double-distilled water) and incubated at 37 °C for 5 h. Piezoelectrically spraying (yellow nozzle, level 4, Derivatizer) of 700 μL of MTT substrate solution (2 mg/mL in PBS buffer) onto the wet plate followed. After a 24-h incubation, the plate was dried (5 min) and detected under Vis (Visualizer 2). Mutagens appeared as purple zones against a light-purple plate background, which resulted from spontaneous mutations on the plate background. Each planar bioassay was performed at least twice to ensure the reproducibility of the bioautograms.

### On-Surface Metabolization Using the S9 Liver Enzyme System

The protocol was identical, with the following exceptions. The positive control was 2AA (0.1 μg/band). Instead of 2.5 mL deficiency medium, the pellet was resuspended either in the ready-to-use solutions of 1870 μL deficiency medium, 505 μL buffer salts, 280 μL S9 fraction, 92 μL NADP, 30 μL buffer M, and 23 μL G-6-P for the rat liver S9,^41^ or in 2400 μL deficiency medium, 25 μL HiCultS9 solution, 25 μL NADP (300 mM), G-6-P (1.5 M), and MgCl_2_ (500 mM) solutions for the human liver S9.^48^ Zone fixation (Degalan solution) was not required, as the S9 enzymes (high-molecular-weight protein structures) enabled sufficient zone fixation on the HPTLC plate.

### Planar SOS-Umu-C Bioassay for Comparison with Genotoxicity

The workflow (requires an S1 laboratory) has already been described.^49,50^ Briefly, the positive control was 4NQO (1 μg/band). The overnight culture of *S.* Typhimurium strain TA1535 was adjusted to an OD_600_ of 0.2 and piezoelectrically sprayed onto the chromatogram (red nozzle, level 4, Derivatizer). The seeded plate was placed in a polypropylene box, as mentioned above, and incubated at 37 °C for 3 h. After the incubation, the plate was dried (5 min). A resorufin-β-D-galactopyranoside substrate solution (2.5 mL of phosphate buffer containing 12.5 μL of the 5-mg/mL stock solution in DMSO) was piezoelectrically sprayed (yellow nozzle, level 5, Derivatizer) and incubated at 37 °C for 30 min. After drying (5 min), the plate was detected at Vis and FLD 254 nm (Visualizer 2).

### Quantitative Dose–Response Analysis

Three dose–response studies (10–1200 μg each), using different cultures, were performed independently on three different plates and days. Videodensitometry was used to obtain the peak areas of mutagen zones in each bioautogram recorded at Vis (formazan absorbance, integrated using the lowest-slope baseline correction and no filter color, VideoScan software version 1.02.00, CAMAG). Mean mutagen zone peak areas (*n* = 3) were used to build the sigmoidal Hill function and calculate the ED_50_ (Python version 3.12.0 within the Anaconda distribution version 2023.07, Anaconda, Austin, USA).

### 2LabsToGo-Eco HPTLC–Duplex Planar Ames–Cytotoxicity Bioassay–Vis with HiCultS9 Metabolization

The 2LabsToGo-Eco analysis was performed as described.^41^ Briefly, the cosmetic or skin care cream extracts were filtered through a syringe filter (pore size 0.2 μm, membrane diameter 25 mm, CHROMAFIL Xtra PTFE, Macherey–Nagel, Düren, Germany) into 2 mL Brand microcentrifuge tubes with a 0.3 mm thin cap (Carl Roth). Solutions (8 μL/band) were applied as 8 mm bands with a distance in between (gap) of 4 mm, from the bottom (offset bottom) 13 mm, and from both sides (offset left/right) 10 mm onto an HPTLC plate silica gel 60 cut to 10 cm × 10 cm and dried (2 min). The settings were: motor speed of 5000 mm/min, initial pressure of 5 psi, frequency of 1000 Hz, distance between drops delta Y 0.01 mm and delta X 0.58 mm, temperature of 100 °C, nozzle of 0.13 mm, and rinsing period of 5 cycles.

The 5 mL syringe was filled with the mobile phase cyclohexane–methyl-*tert*-butyl ether 4:1, *V*/*V* (filtered through a syringe filter as mentioned) and inserted into the syringe–pump system. After placing the glass cover on top of the multifunctional heatable plate holder, the horizontal development up to 65 mm was started by delivering a continuous jet by moving the nozzle along the plate overhang forward and backward. The settings were: syringe 5 mL, syringe load 5.5 mL, rinsing volume 500 μL, motor speed 1 mm/s, motor speed 35 mm/s, initial pressure 15 psi, temperature 0 (off), 0.13 mm nozzle, plate offset left/right −3 mm, offset top/bottom 3 mm, development volume 5 mL, number of passes 84, print both ways marked, and resulting estimated flow rate 20 μL/s.

After plate drying (5 min), chromatogram detection was performed under Vis with the settings: auto exposure on, number of images set to 1, and delay time between images of 0 s. Analogously, 1.7 mL of the *S.* Typhimurium strain TA98 suspension containing the human liver S9 was nebulized (Nebulizer, 11 μm atomizer, intensity 50) onto the HPTLC chromatogram and incubated (Mini-Incubator, 37 °C, 5 h). Then, the 500 μL MTT substrate solution was nebulized, followed by another on-surface incubation (37 °C, 24 h), plate drying (5 min), and analogous bioautogram detection.

## Results and Discussion

### Outline of the New Duplex Planar Ames–Cytotoxicity Bioassay–Vis Including S9 Metabolization

The first attempt at a planar Ames bioassay^45^ needed to be improved regarding the sensitivity and selectivity of detection via the nonselective bromocresol purple pH indicator substrate of the Ames MPF *in vitro* assay,^10,11,44^ as well as zone resolution. A variety of substrates were studied for better selectivity and sensitivity. The best result was obtained with the tetrazolium salt MTT.^53^ The workflow of the newly developed HPTLC–duplex planar Ames–cytotoxicity bioassay–Vis method, including S9 metabolization, is composed (Figure 1) either with zone fixation or metabolization via the human or rat liver S9 enzyme system. Alternative working with ready-to-spray cryostocks^54^ makes handling simple. In detail, after the planar chromatographic separation of up to 22 different samples in parallel, the auxotrophic *Salmonella* in the deficiency medium were sprayed onto the separated samples and incubated for 5 h. On mutagen bands, the *Salmonella* were reverted and turned prototrophic and thus metabolically active during this period. On the plate background, the auxotrophic *Salmonella* in the deficiency medium died or were severely inhibited in their metabolism. The first 5 h of incubation (brightening the background) was a precondition for the second 24 h of incubation with the MTT substrate applied on the same surface. After optimization of the MTT substrate amount on the plate (Figure S1A), the hindered metabolism of auxotrophic *Salmonella* after the first 5-h incubation provided a bright plate background and thus made detectable the few spontaneous reversions in the second 24-h incubation visible as a light purple plate background. In contrast, mutagens in the separated samples caused many reverse-mutated *Salmonella*, which transformed to be prototrophic and metabolically active, and thus intensely reduced the MTT substrate to the purple formazan. Thus, a purple band indicated the presence of a mutagen. Note that the formazan coloration was pH-dependent: *i.e*., purple at pH 3 *versus* reddish orange at pH 9 (Figure S1B). In addition, the low rate of spontaneous reverse mutations in the *Salmonella* population resulted in a faint light-purple background signal across the plate. Whitening yellowish zones, where these minimal spontaneous reversions were no longer detectable, thus reflected a strong cytotoxic effect due to severe inhibition of cellular metabolism along with cell death. The whitening response is therefore operationally interpreted as a cytotoxic endpoint in addition to the purple mutagenicity read-out. This highlighted the key advantage of simultaneously visualizing and differentiating individual mutagenic and cytotoxic compounds within the same separated sample on a single bioautogram. This selective dual read-out of mutagenicity and cytotoxicity via cell viability was considered a novel approach and a great benefit. It improved the efficiency and reliability of the Ames bioassay. Interesting aspects of bioassay development are discussed as follows.

**Figure 1:**
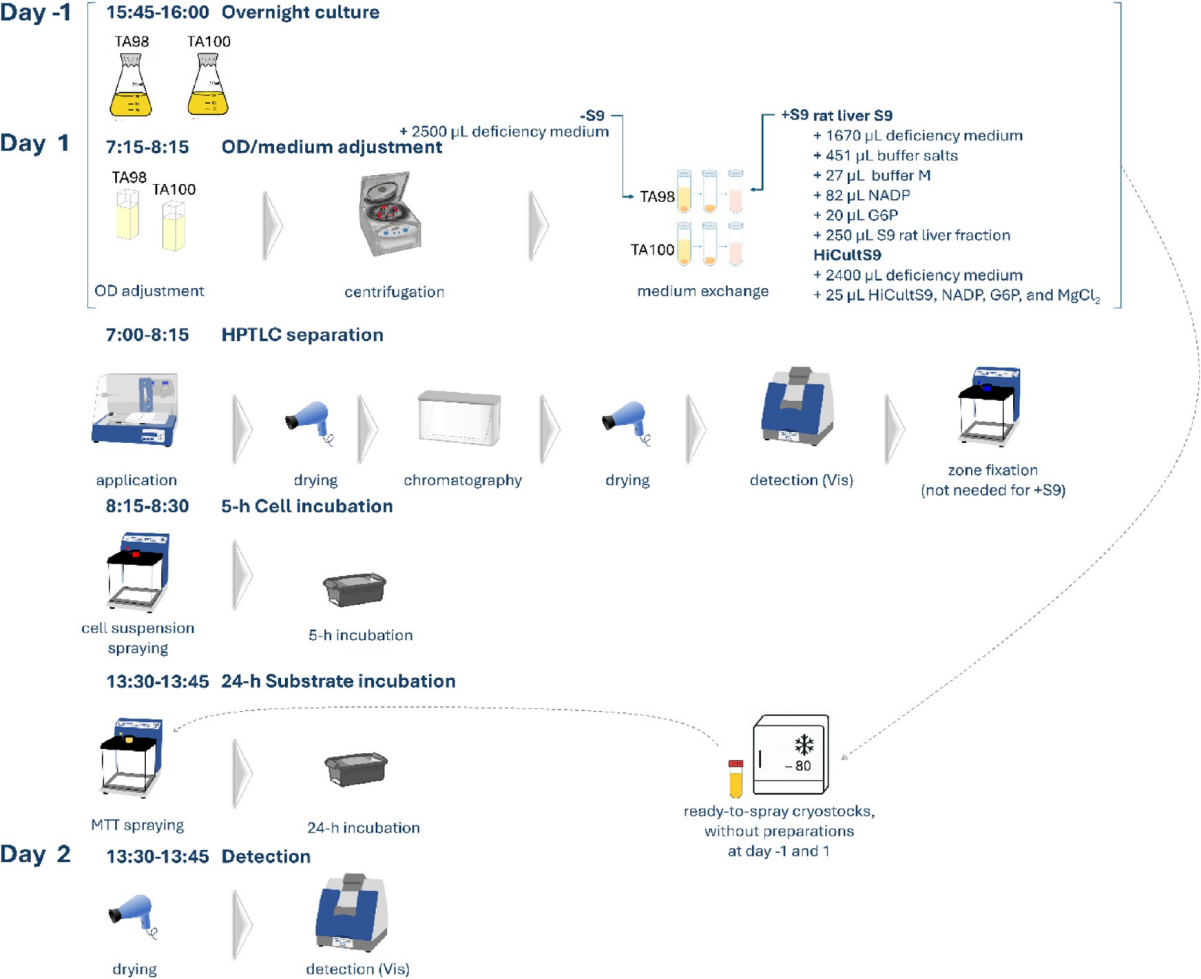
Routine workflow of the newly developed HPTLC–duplex planar Ames–cytotoxicity bioassay–Vis method, either with zone fixation or metabolic S9 activation via the human or rat liver S9 enzyme system, manual operation time summed up to 3.3 h; 4 plates (88 samples) can be screened in parallel per 8-h working day (9-h day including breaks). On the day before/after, work takes only 15 min per plate, which can easily be integrated into the daily schedule; alternative working with ready-to-spray cryostocks^54^ nihilates the 1.3-h works such as OD adjustment on day 1, and makes handling simple.

### Testing of Mutagenic Positive Controls

The initial duplex planar Ames-cytotoxicity bioassay did not use zone fixation, and the second on-surface incubation was 25% shorter compared to the final protocol (18 h instead of 24 h). Different amounts of three mutagenic positive controls and three further ones requiring metabolic activation^46^ were tested with/without *Salmonella* strains TA98 and TA100 and with/without the rat liver S9 enzyme system (Figure 2). The bacteria-free control plates and the prebioassay Vis images verified that the purple color formation (reduction of MTT to the purple formazan) was specifically caused by the reverse mutation of the *Salmonella*. The 4NQO produced a mutagenic response in TA98 at 0.3 μg/band (purple inner core zone), while TA100 showed no detectable mutagenicity for the tested 4NQO amounts. This demonstrated strain-dependent mutagenicity: TA98 detected frameshift mutations via hisD3052 reversion targeting GC-repeat regions, whereas TA100 detected base-pair substitutions via reversion at a specific GC site in the hisG46 gene.^4,5^ For N4ACT, mutagenicity was observed at 1 μg/band for TA98 and TA100. The DMSO, though primarily used for dissolution and thus as a solvent control, exhibited mutagenicity at higher test amounts (200 μg/band for TA98; 1000 μg/band for TA100). While DMSO is not conventionally considered mutagenic, these observations corroborate reports of specific mutagenic effects on *Salmonella* strains.^55,56^ Such effects were observed only at concentrations higher than those present in the Ames MPF *in vitro* assay, which typically uses DMSO as the solvent for mutagenic positive control standards.^11^ In the latter *in vitro* assay, DMSO dilution and solvent controls effectively account for potential solvent-related mutagenicity, which is unlikely to be detectable under these conditions. However, mutagenic impacts cannot be completely excluded.

Requiring metabolic activation,^46^ the AO induced a mutagenic response in both strains at 0.001 μg/band via the rat liver S9 enzyme system and a strong mutagenic response in both strains at 0.01 μg/band via the human liver S9 enzyme system, but this was masked by its own yellow–orange color at higher amounts of 0.1 μg/band (rat liver S9 enzyme system) and 1 μg/band (human liver S9 enzyme system), already detected in the prebioassay Vis image, though weaker. The 2AA showed only minor strain-specific differences, *i.e*., arising mutagenicity at 0.001 μg/band, including the rat liver enzyme system, which was slightly better detectable via TA100 than TA98. Similar zones on the *Salmonella*-free negative control plate (marked – ), with metabolic activation via the rat liver enzyme system (+Rat liver S9) are explained by S9 reductases triggered by 2AA, which cause a false-positive cytotoxic/mutagenic response at amounts above 1 μg/band. Hence, a *Salmonella*-free negative control plate is recommended for verification of metabolic activation (+Rat liver S9). Testing the 2AA with the human liver S9 enzyme system, such effects are not detectable due to differences in its composition (Figure S2). 2AA induced a stronger mutagenic effect via the human liver S9 enzyme system at 0.01 μg/band. The 2AF at 0.001 μg/band displayed mutagenic activity via the rat liver enzyme system in both strains. However, it was masked by its yellow–orange color at amounts above 1 μg/band, already detected in the prebioassay Vis image, though weaker. Testing 2AF via the human liver S9 enzyme system, a stronger mutagenic response was detectable in both strains at 0.01 μg/band. The overall mutagenicity via metabolic activation down to 1 ng/band was found to be sufficiently sensitive for the following sample screening. Positive controls (Figure S2) were applied to each subsequent bioautogram to prove and ensure the proper functioning of the bioassay.

**Figure 2:**
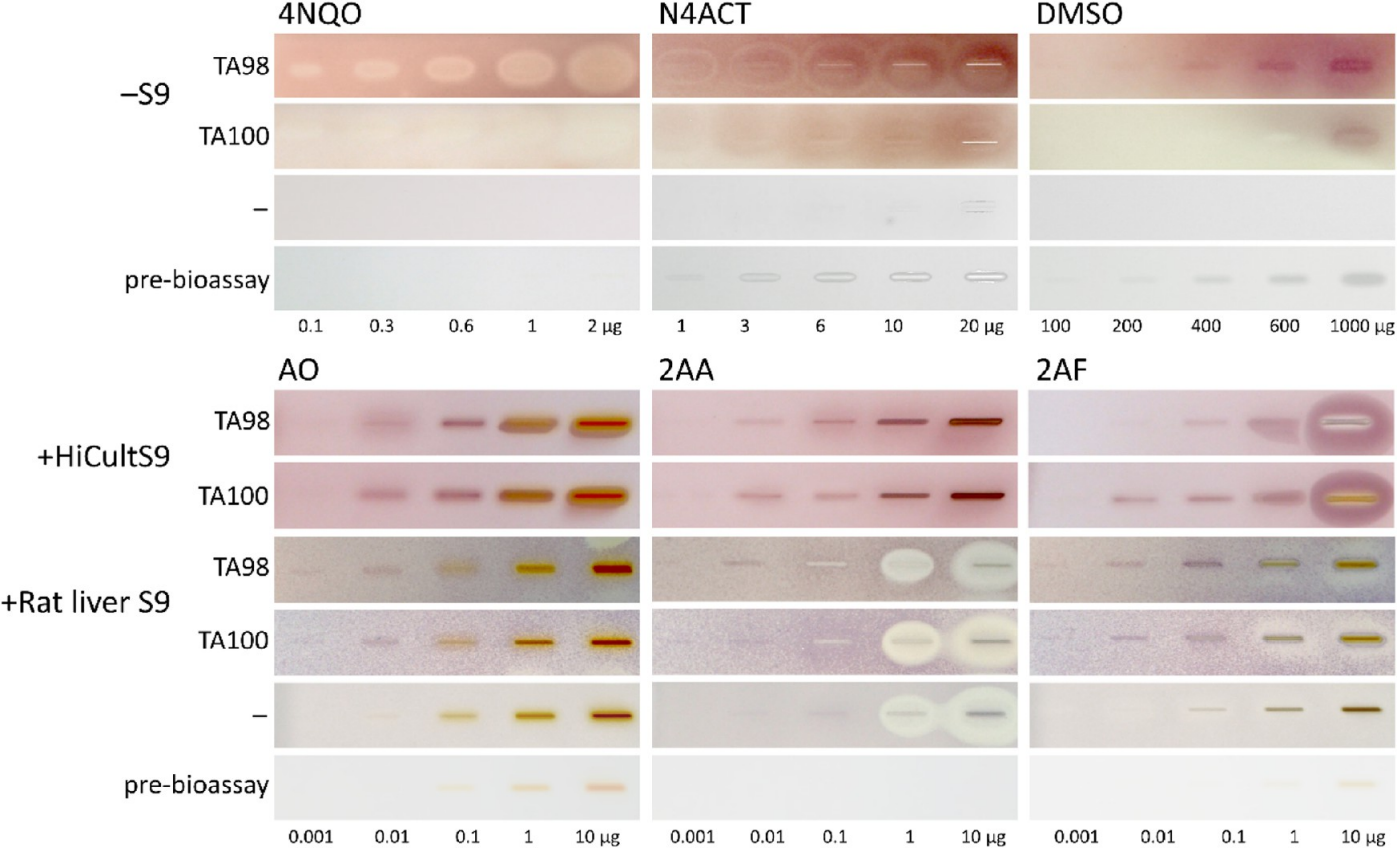
Six different mutagenic positive controls, *i.e*., 4NQO (0.1–2 μg), N4ACT (1–20 μg), and DMSO (100–1000 μg), and for metabolization with the rat liver S9 and HiCultS9 enzyme system, AO (0.001–10 μg), 2AA (0.001–10 μg), and 2AF (0.001–10 μg), were applied and detected via the initial (no zone fixation and 25% shorter second incubation) duplex planar Ames–cytotoxicity bioassay–Vis with the *Salmonella* strain TA98 or TA100 without (−S9)/with (+S9) metabolization, and for comparison, negative control plate without a strain (marked –; the rat liver S9 enzymes cause a whitening or its reductases an MTT reduction; both is not evident for the HiCultS9 in Figure S2) and prebioassay Vis plate (showing native colors for high amounts).

### Application to Complex Samples

Microtiter plate-based Ames assays are unable to deal with the high complexity of complex samples, such as tea drugs. They assess only the overall activity of a sample as a sum value, which makes the assay endpoint highly susceptible to matrix effects. In contrast, the planar bioassay enables chromatographic separation as well as detection, differentiation, and assignment of individual mutagens, independent of the matrix. Such a combination of separation with selective and sensitive detection can be applied to cope with sample complexity. Thus, the initial (no zone fixation and 25% shorter second incubation) duplex planar Ames-cytotoxicity bioassay was applied to methanolic extracts of five different tea sorts (Figure 3). The MTT substrate and a longer second incubation (18 h instead of 5 h) performed better than the first attempt of a planar Ames bioassay with the bromocresol purple pH indicator substrate and a short 5-h incubation.^45^ Distinct purple mutagen zones were detected in black (1, *e.g.,* zones **a**–**c**) and herbal tea extracts (4 and 5, *e.g.,* zone **i**). These zones were absent on the respective *Salmonella*-free negative control plate, verifying the mutagenic response. Some mutagens remained stable after metabolization via the rat liver S9 enzyme system (5, *e.g.,* zone **l**), suggesting metabolically resistant mutagens. Other mutagens (4, *e.g*., zone **j**) disappeared after S9 metabolization via cytochrome P450-mediated reactions, mainly Phase I oxidations along with carbon hydroxylation, heteroatom oxygenation and dealkylation, epoxidation, and a variety of other transformations, yielding nonmutagenic metabolites that are no longer detectable, indicating detoxification.^57,58^

On both *Salmonella*-free negative control plates with/without metabolization via the rat liver S9 enzyme system, in particular, the green tea extracts caused not only enzymatic S9-induced but also chemical MTT reduction (Figure 3),^59^ explained by catechins acting as redox-active compounds.^14^ This highlights the need for *Salmonella*-free negative control plates to allow a sound interpretation of mutagens detected in polyphenol-rich samples. To support the MTT-based findings, the genotoxicity of the same tea sort extracts was additionally evaluated using the HPTLC–SOS-Umu-C–FLD bioassay (Figure S3). Similar zone-specific genotoxic effects were observed, with notably stronger fluorescence signals after S9 activation. As demonstrated by these reliable results and the important information obtained for highly complex samples, the planar bioassay format offers a decisive advantage over classical *in vitro* assays. It resolved multiple distinct mutagenic and cytotoxic compound zones in a highly complex matrix, which typically masks individual responses in a microtiter plate assay format.

### Use of Zone Fixation, Shown for Cosmetics and Skin Care Cream Extracts

Zone sharpness and, thus, resolution were compromised by zone diffusion during the long incubation at high humidity. Hence, the improvement in zone sharpness using zone fixation via Degalan^60,61^ was studied for the analysis of mutagens in 22 cosmetics and skin care creams, as recently shown for genotoxicity.^41^ Mutagens (Figure 4, purple zones **a**–**v** labeled in the first extract, standing for further extracts) were detected in almost all extracts. Using a 0.5% Degalan concentration reduced zone diffusion, resulting in sharper zones and enabling clear visualization of the purple mutagen zones. Potentially reduced signal intensities due to excessive Degalan on the surface were not observed. Degalan formed a polymer film that efficiently fixed the substance zones while still allowing sufficient diffusion of the substances in the aqueous medium, ensuring contact with the *Salmonella* cells. Thus, the polymer film reduced diffusion without impairing the bioassay response, as evidenced by comparable signal intensities in sample zones with and without zone fixation.^60,61^ Zone fixation with 0.5% Degalan was used for all subsequent bioautograms (marked as HPTLC^fix^), except for plates with S9 metabolization, for which the large S9 enzyme proteins provided sufficient zone fixation, making additional polymer film fixation unnecessary.

**Figure 3:**
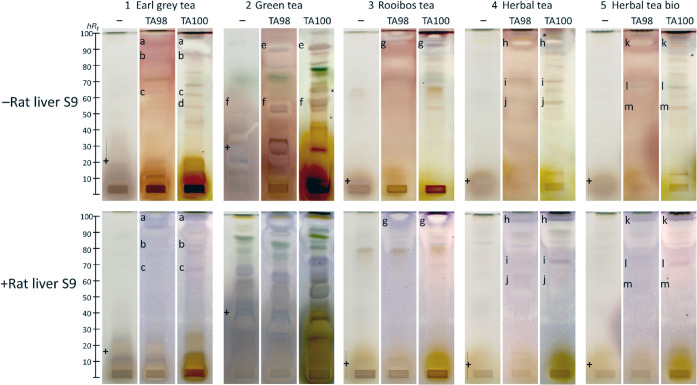
Mutagenicity/cytotoxicity bioautograms of the different methanolic tea extracts 1–5 (3 mg/band, Table S1, separated on HPTLC plate silica gel 60 with dichloromethane–methanol–ammonia 85:15:1, *V*/*V*/*V*) showing purple mutagen zones **a**–**m** and whitening yellowish cytotoxic compound zones via the initial (no zone fixation and 25% shorter second incubation) HPTLC–duplex planar Ames–cytotoxicity bioassay–Vis using *Salmonella* strain TA98 or TA100 without (−S9) and with (+S9) metabolic activation; verification of the bioassay response by the *Salmonella*-free negative control plate (false positives +) and by positive controls (Figure S2).

### Prolongation of the Second Incubation, Shown for Cosmetics and Skin Care Cream Extracts

To enhance detection sensitivity, the second 18-h incubation time was extended to 24 and 48 h, which was tested for both *S.* Typhimurium strains TA98 and TA100. On the one hand, the 18-h incubation already detected the major mutagens; however, it failed to detect the weak mutagens, as the purple formazan coloration was comparatively weaker for 18 h (Figure 5, *e.g.*, zone **b** in 7–9 and zone **d** in 2 and 3, labeled in the first extract, standing for further extracts). Notably, for TA100, signals were comparatively weaker for an incubation of 18 h than 24 h (*e.g.*, TA100 zone **c** in 2–10 and zone **k** in 5). On the other hand, extending the incubation to 48 h resulted in higher zone diffusion in several cosmetic and skin care cream extracts. Overall, the 24-h incubation represented a good compromise between signal intensity and zone sharpness. It ensured comprehensive detection of mutagens while maintaining zone resolution and was thus selected for all subsequent bioassays.

### Interpretation of Mixed Zone Appearance

Mixed zone appearances were evident in the bioautograms. This phenomenon was most pronounced for lipophilic samples, caused by coelution of candidates of the same structural group, such as the different MAGs, DAGs, and TAGs. Exemplarily, zone **b**, which was assigned to DAGs, is discussed in the following. On the one hand, an intense purple mutagen center zone was surrounded by a sharp whitening yellowish halo (*e.g*., Figure 5, 24 h, zone **b** in 7 and 9). The purple center zone indicated a mutagen, while the whitening yellowish halo (no cell metabolism) suggested a coeluting cytotoxic compound. On the other hand, a whitening yellowish cytotoxic compound zone center was enclosed by a purple rim (*e.g*., 24 h, zone **b** in 10), which was explained by a coeluting mutagen in the rim. In addition, another mutagen in the zone center (*e.g*., TA98, 24 h, zone **b** in 5) coeluted with a surrounding whitening yellowish cytotoxic compound zone and was enclosed by a mutagen evident as a purple rim. Dose–response studies of sample 5 using TA98 and TA100 showed that the appearance of zone **b** depended on the amount applied, forming the mixed zone appearance with increasing amounts (Figure S4).

**Figure 4:**
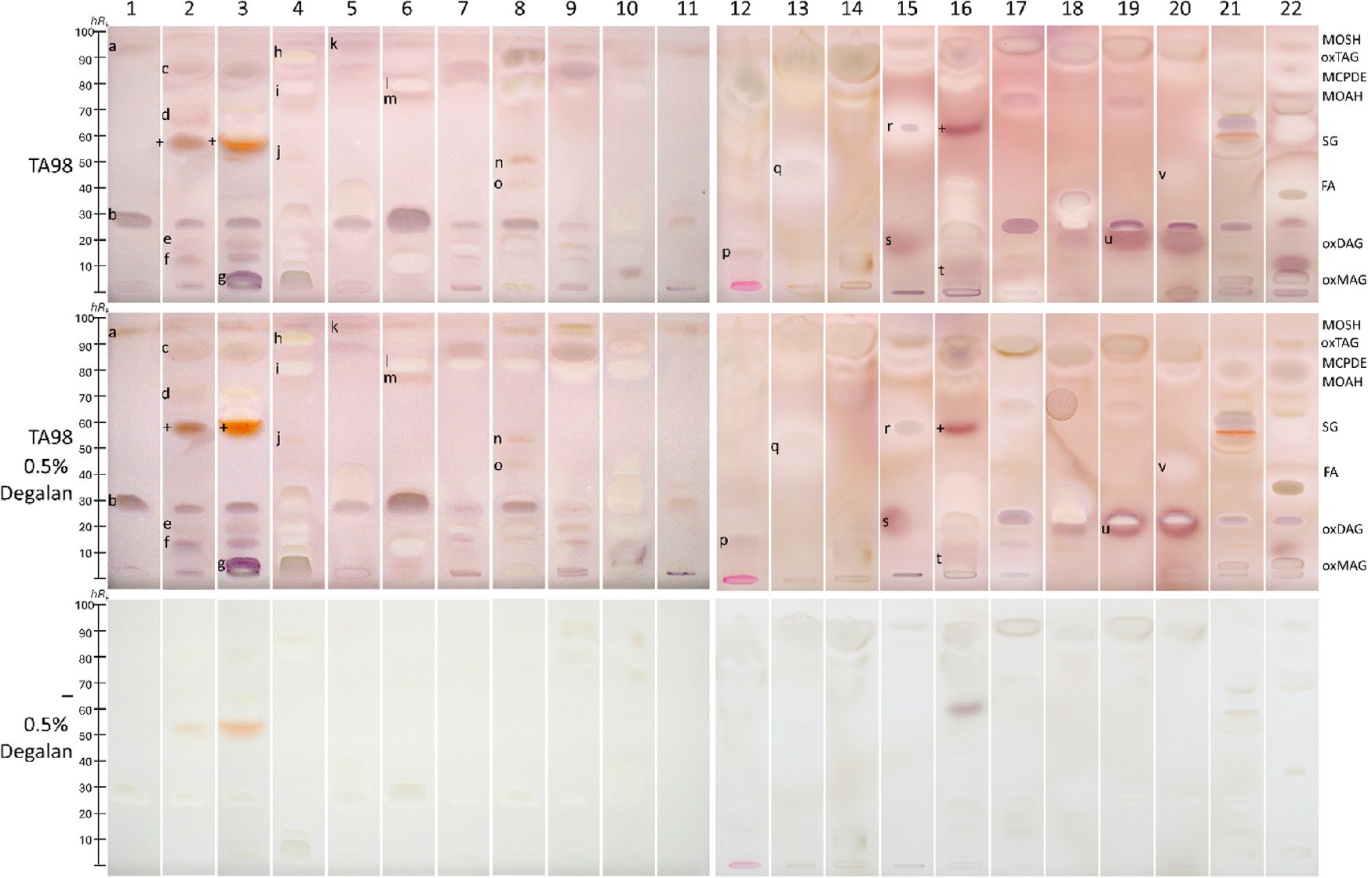
Zone fixation study: Mutagenicity/cytotoxicity bioautograms of the different cosmetic and skin care cream extracts 1–22 (Table S1, 0.8 mg/band each, separated on HPTLC plates silica gel 60 with pentane–diethyl ether 8:3, *V*/*V*), showing purple mutagen zones **a**–**v** and whitening yellowish cytotoxic compound zones (assigned according to Figure 7) via the initial (no zone fixation and 25% shorter second incubation) HPTLC–duplex planar Ames–cytotoxicity bioassay–Vis with *Salmonella* strain TA98 and with/without zone fixation using 0.5% Degalan. Verification of the bioassay response by the *Salmonella*-free negative control plate (false positives +) and by positive controls (Figure S2).

The individual candidates in the mixed zone appearance can be clarified by testing an optimal mobile or stationary phase for dose-dependent migration (the current focus was on screening), or by a highly streamlined hyphenation,^60,62,63^ *i.e.*, an online orthogonal second separation (reversed-phase, *e.g*., RP-C18 phase) can clarify which candidates are coeluting in the same zone. Directly from the bioautogram and fully automated,^64^ the active compound zone can be further characterized by elution to RP-HPLC, diode-array detection (DAD), and HRMS/MS. The compound zone was eluted together with the salt-rich bioassay media from the silica gel layer (normal phase, NP), trapped on a precolumn in a loop during desalting via a valve switch, and then directed to orthogonal RP-HPLC–DAD–HRMS/MS. Using NP-HPTLC–UV/vis/FLD–EDA–RP-HPLC–DAD–HRMS/MS, up to 12 different physicochemical, chromatographic, effect-directed, and spectro­(photo)­metric features about the unknown active compound zone can be obtained, as demonstrated by highly efficient 8–,^63,65^ 10–,^62,66–69^ and 12–dimensional hyphenations.^60^ The more information the scientist gains, the faster the identification of compounds.

### Metabolization via Rat *versus* Human Liver S9 Enzyme System

The comparison of metabolization via the rat liver S9 enzyme system between strains TA98 and TA100 showed hardly any differences in the mutagen zone patterns (Figure 6A *versus* [Fig fig6]B). For both strains, the same mutagen zones were detected, indicating that the same substances were activated to mutagens. The mutagenic nature of the observed signals was verified by a respective *Salmonella*-free negative control plate with the S9 enzyme system, which showed no purple compound zone except for one faint brownish false positive zone (Figure 6A, marked + in 2 and 3). This faint brownish compound zone also appeared on the S9-free and *Salmonella*-free plate (Figure 4), whereas it did not appear on the prebioassay Vis chromatogram (Figure S5) and thus was caused by chemical MTT reduction. Hence, the corresponding mutagen zone (+) is slightly overrated in its mutagenicity by this faint brownish response. Several mutagen zones maintained their stable purple coloration with *versus* without metabolization, indicating unchanged mutagenicity (Figure 6A *versus* [Fig fig6]B, *e.g*., zone **b** in 1–3). Altered toxification was also observed for whitening yellowish cytotoxica (*e.g*., zone **l** in 6–8 and zone **h** in 4), which shifted to a subcytotoxic but mutagenic compound amount visible as a peripheral purple rim after metabolization. The individual active zones were better detectable on the subcytotoxic S9 bioautogram (*e.g*., zone **o** in 10). The question of whether cytotoxicity or mutagenicity was the worse option remains. Other weak mutagen zones increased in mutagenicity after metabolic activation (*e.g*., zones **e** and **f** in 2 and 9, zone **c** in 2, 3, 7, and 9, and zones **n** and **o** in 8), *i.e*., the promutagenic compounds required enzymatic conversion to their mutagen form, indicating toxification. Such an S9-dependent metabolic activation through cytochrome P450 enzymes is known for oxidizable lipophilic compounds, including unsaturated fatty acid derivatives, glycerides, or mineral oil residues^70–73^ capable of inducing point mutations in bacterial systems by electrophilic intermediates, such as epoxides, peroxides, and α,β-unsaturated carbonyls.^74^

**Figure 5:**
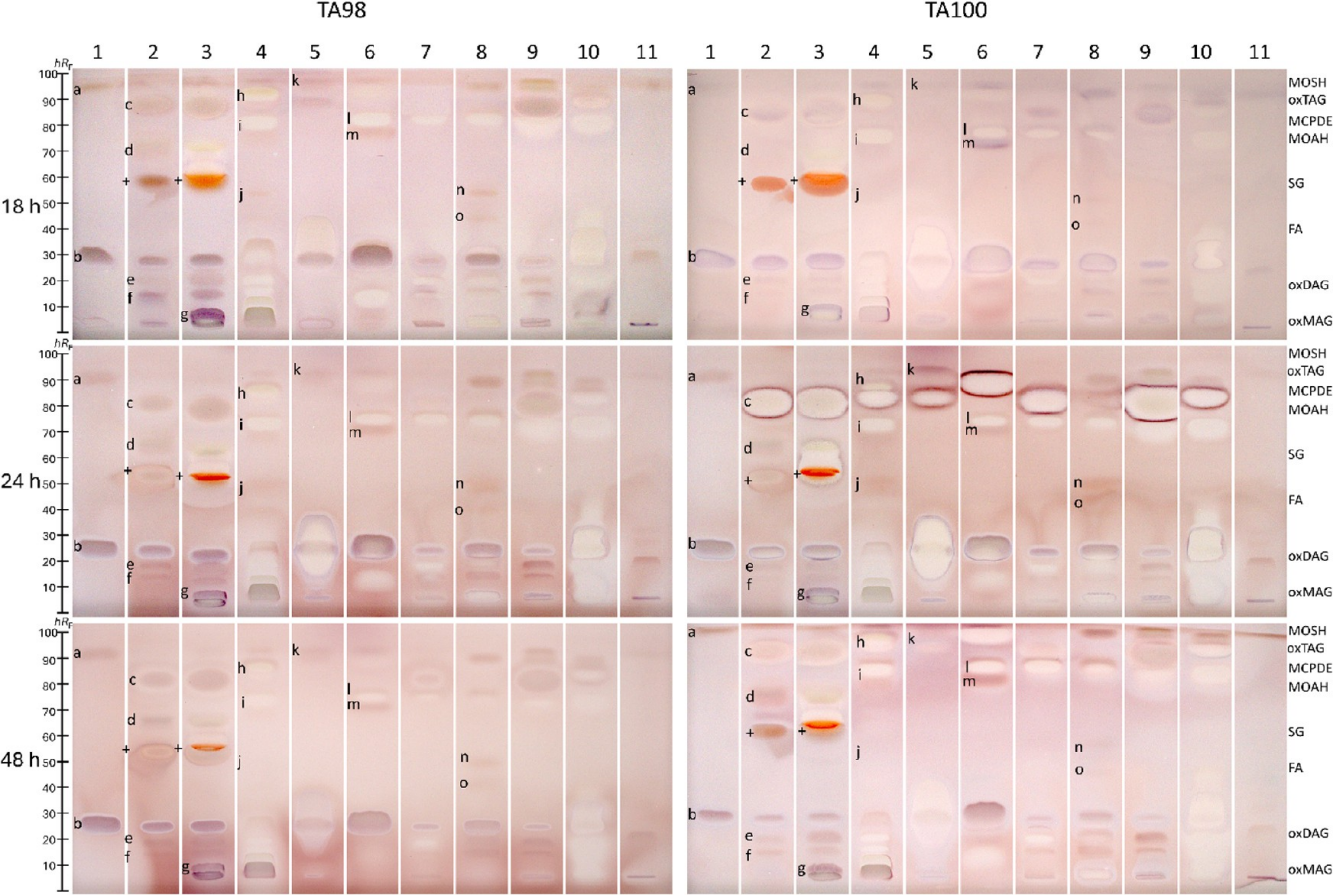
Study of a prolonged second incubation time: mutagenicity/cytotoxicity bioautograms of the different cosmetic and skin care cream extracts 1–11 (Table S1, 0.8 mg/band each, separated as in Figure 4) showing purple mutagenic substance zones **a–o** and whitening yellowish cytotoxic compound zones (assigned according to Figure 7) via the HPTLC^fix^–duplex planar Ames–cytotoxicity bioassay–Vis using *Salmonella* strain TA98 or TA100 and incubation times of 18, 24, and 48 h; zones showed higher *hR*_F_ values on the 18-h bioautogram (higher humidity of the surrounding air slightly deactivated the adsorbent activity). Verification of the bioassay response by positive controls (Figure S2).

Species-dependent metabolization was studied, and the results were compared. Both the rat and human liver S9 enzyme systems can activate a broad spectrum of promutagens; however, the composition and expression (quantities) of cytochrome P450 enzymes differ.^57^ The human liver S9 enzyme system more closely reflects human physiology and activity of cytochrome P450 enzymes,^75^ and thus, it was tested with a new animal-free cultivated, cell line-derived human S9 fraction (HiCultS9).^76,77^ After metabolic activation with the HiCultS9,^48^ the TA98 bioautogram showed stronger purple mutagen zones (Figure 6C) than the counterpart using the rat liver S9 enzyme system (Figure 6A), and thus, mutagenicity increased by simulated human metabolization. Although the metabolic activation of the mutagens in the 11 different cosmetic and skin care cream extracts was more intense for HiCultS9, both liver S9 enzyme systems produced similar mutagen profiles. Such species-dependent metabolic activation, differing in intensity and expression, was already reported.^58,78^ The use of the human liver S9 enzyme system (HiCultS9) increased the reliability of toxicological assessment; however, unfortunately, it resulted in even more pronounced mutagenicity.

**Figure 6:**
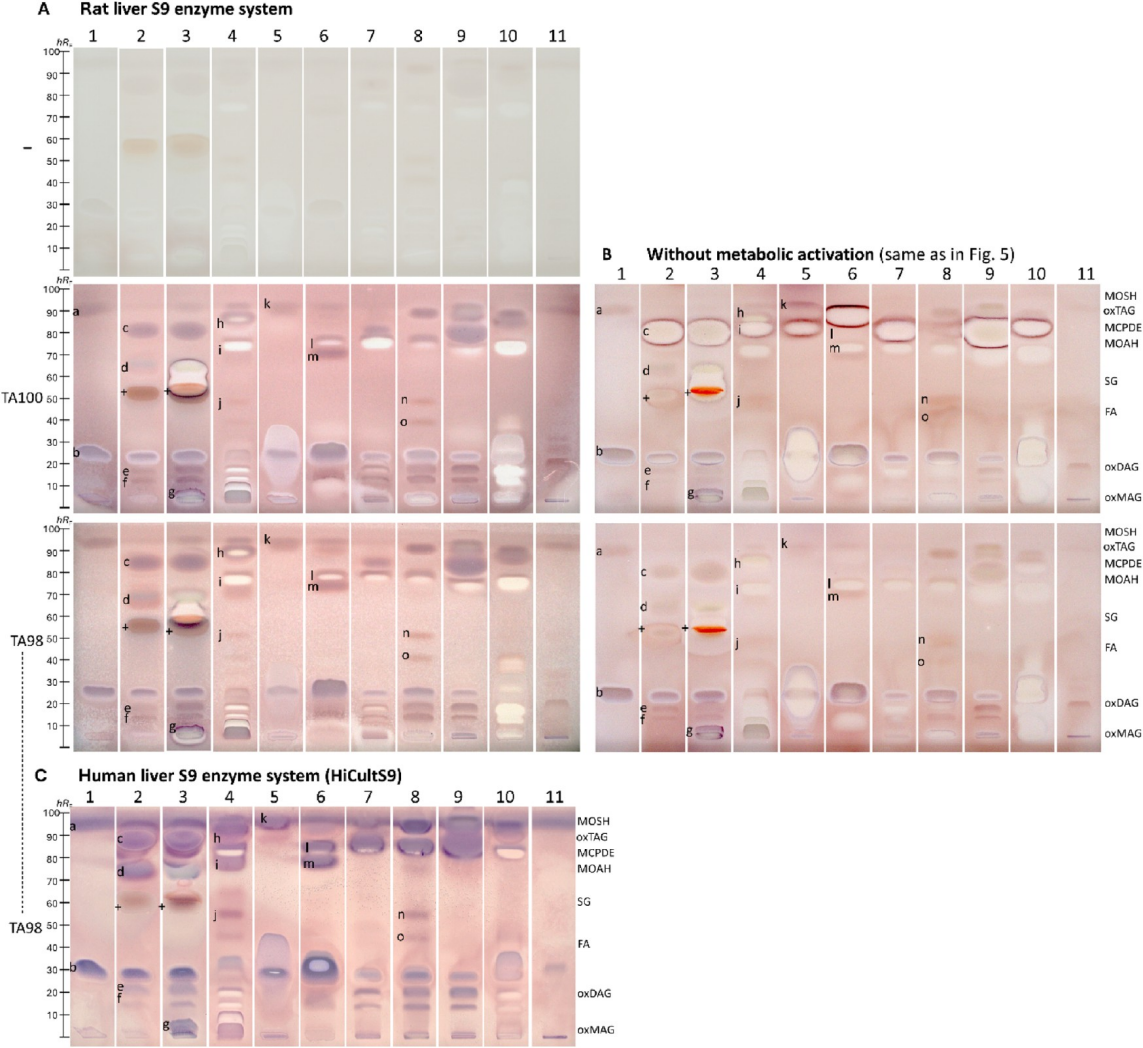
Species-dependent metabolization: mutagenicity/cytotoxicity bioautograms with metabolization via the rat (**A**) *versus* human liver S9 enzyme system (**C,** HiCultS9) *versus* (**B**) without metabolization (Figure 5, 24-h incubation) of the different cosmetic and skin care cream extracts 1–11 (Table S1, 0.8 mg/band each, separated as in Figure 4) showing purple mutagenic substance zones **a–o** and whitening yellowish cytotoxic compound zones (assigned according to Figure 7) via the HPTLC–duplex planar Ames–cytotoxicity bioassay–Vis using *Salmonella* strain TA98 or TA100; zones showed higher *hR*_F_ values on the HiCultS9 bioautogram (a higher humidity of the surrounding air slightly deactivated the adsorbent activity). Verification of the bioassay response by the *Salmonella*-free negative control plate (false positives +) and by positive controls (Figure S2).

### Assignment of Mutagens via Reference Compounds

To characterize the mutagens and cytotoxica detected in the planar bioautogram, HPTLC plates were derivatized with the primrosel reagent and detected at FLD 366 nm (Figure S6). The primulin reagent physicochemically attaches to lipophilic molecules, including TAGs, waxes, and other nonpolar metabolites.^79^ Mutagen zones consistently exhibited blue fluorescence, indicating that the detected mutagens either belong to or comigrate with such lipid classes. Traces of oxidized lipids, including highly potent hydroperoxides and epoxides derived from coeluting unsaturated longer-chain acyl chains or fatty acids in lipid structures, exhibited genotoxicity due to their high reactivity with DNA, as recently revealed by the planar SOS-Umu-C bioassay.^41^ Hence, for a tentative structural group assignment of the mutagens and cytotoxica detected in the extracts, 12 different lipophilic reference compounds were coanalyzed and compared with three cosmetics and skin care creams, *i.e*., 2, 3, and 10, in the planar bioautograms tested with metabolization by the human liver S9 enzyme system (HiCultS9) and without (Figure 7).

**Figure 7:**
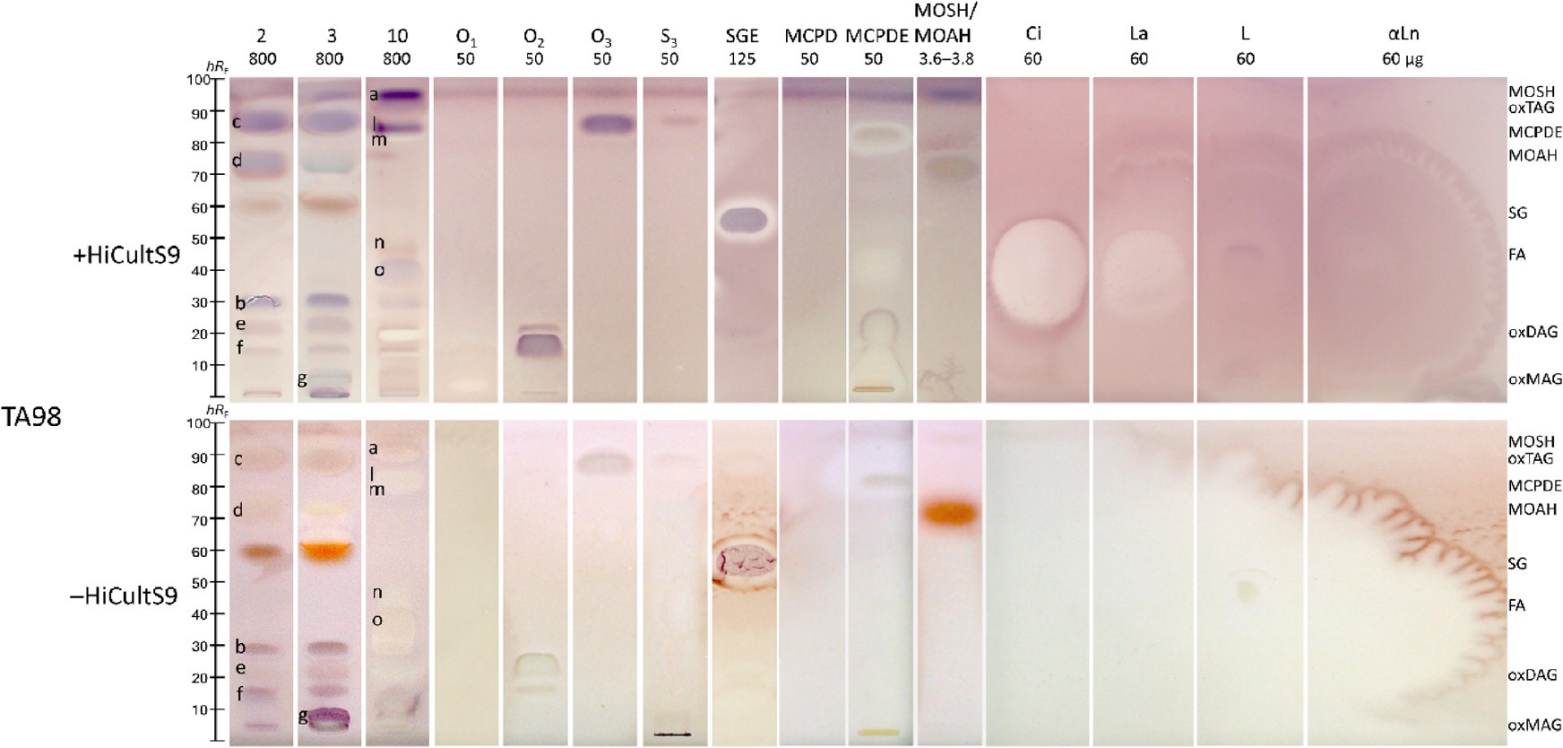
Mutagenicity/cytotoxicity bioautograms with metabolic activation via HiCultS9 and without the three cosmetics and skin care creams 2, 3, and 10 (Table S1, 0.8 mg each, separated as in Figure 4) coanalyzed with 12 different reference compounds (MAG candidate O_1_, DAG candidate O_2_, TAG candidates O_3_ and S_3_, all 50 μg each; SGE, 125 μg; fatty acids Ci, La, L, and αLn, all 60 μg each; MCPD and MCPDE, 50 μg each; MOSH/MOAH mixture, 3.6–3.8 μg) via the HPTLC^fix^–duplex planar Ames–cytotoxicity bioassay–Vis using the *Salmonella* strain TA98, showing purple mutagen and whitening yellowish cytotoxic compound zones. Verification of the bioassay response by positive controls (Figure S2).

The MAG candidate O1 (*hR*_F_ 4) showed a weak indication of mutagenic activity at 50 μg/band in the bioautograms with HiCultS9. Without metabolic activation, however, no mutagenic effect was detectable, which can be explained by the relatively low absolute amount applied per band (50 μg), as a genotoxic response in the SOS-Umu-C assay was observed at 100 μg.^41^ The mutagen zone **g** (Figure 7 in 3 and Figure 6 in 3, 4, 6, and 10) had a similar *hR*_F_ and was assigned to this structural group, supposedly oxidized (ox)­MAGs. The two isomers of the respective DAG candidate O_2_ (*hR*_F_ 14–25) showed, at the same amount, comparatively strong mutagenicity and cytotoxicity in the bioautograms with and without HiCultS9, respectively. The mutagen zones **f** and **e** (Figure 7 in 2 and 3 and Figure 6 in 2–4 and 7–9) and zone **b** (Figure 7 in 2 and 3 and Figure 6 in 1–3 and 4–9) aligned closely with this structural group, most likely oxDAGs. The mutagenic and cytotoxic zone **o** (Figure 7 in 10 and Figure 6 in 4, 8, and 10) in the bioautograms with and without HiCultS9, respectively, corresponds to the fatty acid L (*hR*_F_ 40). The fatty acids (Ci, La, L, and αLn, 60 μg each) showed pronounced cytotoxicity and diffusion in the bioautogram without HiCultS9, whereas an altered toxification toward mutagenicity was observed in the bioautogram with HiCultS9. The SGE (125 μg) and MCPDE (50 μg) revealed strong mutagenicity and cytotoxicity in the bioautograms, but not the MCPD, however, tested at 50 μg only. The mutagen zone **d** (Figure 7 in 2 and Figure 6 in 2 and 3) was tentatively assigned to the MOAH group (candidate perylene at *hR*_F_ 75), especially detected in the bioautograms with HiCultS9. The mutagen zone **l** (Figure 7 in 10 and Figure 6 in 6–8 and 10) and zone **c** (Figure 7 in 2 and Figure 6 in 2, 3, 7–9, and 10) correspond well to the TAG group (triolein and tristearin candidates at *hR*_F_ 88), supposedly oxTAGs, especially detected in the bioautograms with HiCultS9. The mutagen zone **a** (Figure 7 in 10 and Figure 6 in 1–4 and 6–10) matched well with the MOSH candidate 5-α-cholestane (*hR*_F_ 95), especially detected in the bioautograms with HiCultS9. The metabolically resistant mineral oil compounds also persist in human tissues, thereby amplifying their long-term mutagenic potential.^80^ The HPTLC–duplex planar Ames–cytotoxicity bioassay, including S9 metabolization, detected the mutagenic effects of the reference substances at equivalent amounts to those detected for the HPTLC–planar SOS-Umu-C–FLD bioassay (Figure S7). These oxidized lipid-related and MOSH/MOAH-like candidates can form traces of highly potent electrophilic oxidation products (*e.g*., epoxides, peroxides, and reactive carbonyls derived from coeluting unsaturated longer-chain candidates) that are capable of DNA interaction and thus explain the purple mutagen zones observed in the duplex planar Ames-cytotoxicity bioautograms.

### Quantitative Evaluation

Almost all tested 22 cosmetic and skin care cream extracts (Table S1, Figure 4) contained mutagens and/or cytotoxica. To measure the impact, a quantitative evaluation was performed for skin care cream extract 1, representing well the tested products. Three dose–response studies (10–2000 μg/band, *n* = 3) were performed independently on three plates and days using different cultures. A dose-dependent increase in mutagenicity (purple zone tentatively assigned to oxDAGs) of skin care cream extract 1 was observed (Figure 8). Associated cytotoxicity, evident by zone whitening, increased at higher amounts from 800–2000 μg/band. The three mutagenicity bioautograms were evaluated by videodensitometry. The mean dose–response curve was built, and the ED_50_ value was calculated to be 677 μg. The ED_50_ value is estimated to be even lower, given the incomplete extraction of mutagens from the skin care cream. The associated cytotoxicity lowered the mutagenicity signal (whitening of the purple signal) and caused the curve to flatten at high amounts.

Daily application amounts for different skin care products were estimated at 7.8 g for body lotion, 2.2 g for hand cream, and 1.5 g for face cream.^81^ The daily exposure may even exceed the summed consumption of 11.5 g for products used concurrently and multiple times per day. Already, the exposure to an exemplary 11.5 g of skin care products corresponds to an ED_50_ exceedance of approximately 17,000. This means that the amount required to elicit a half-maximal mutagenic response in the Ames assay is exceeded by at least 4 orders of magnitude for the daily use of skin “care” products. The detection of mutagenic activity at submilligram levels is particularly concerning, as it indicates that even low amounts may pose a risk of mutagenicity. Quantitative dose–response analysis, such as the determination of ED_50_, is therefore valuable for risk assessment. It is widely recognized that no safe threshold can be assumed for mutagens.^82^ Mutagens can enter the body via microinjuries with open channels to the blood circuit, *e.g*., those caused by shaving body hair. The new mutagenicity results call for a reflection and reevaluation of the risks of daily use of cosmetic and skin care products and a precautionary or proactive consumer protection, which currently does not exist, as recently discussed.^1,38,41^

### Further Application to Perfumes

Unlike lipophilic cosmetic and skin care cream extracts, perfumes represent a separate class of volatile and compositionally diverse substances. Without sample preparation (since perfumes are already a liquid), perfumes (1 μL each) were directly analyzed using the HPTLC^fix^–duplex planar Ames-cytotoxicity bioassay–Vis method. With both *S.* Typhimurium strains TA98 and TA100, distinct purple mutagen and whitening yellowish cytotoxic compound zones were observed (Figure 9). All purple mutagen zones were absent on the *Salmonella*-free negative control plate, verifying that MTT was reduced solely due to bacterial metabolic activity. Supporting strain-specific mutation, zone **a** in 1, zone **e** in 3, and zone **o** in 8 were detected to be mutagenic with TA98 (frameshift mutations), whereas the same amounts were already cytotoxic with TA100 (base-pair substitutions). The mixed zone appearance was already discussed, as evident for zones **b**, **d**, **j**, and **l** (whitening yellowish cytotoxic compound zone center with a mutagen purple rim), and zone **n** (purple mutagen center surrounded by a whitening yellowish halo with a faint purple rim). Several mutagens and cytotoxica were detected in only 1 μL of perfume. Given a 100-μL pump spray of perfume, the cytotoxic and mutagenic effects would be 100-fold more intense on the skin, as detected in the bioautogram. Furthermore, metabolic activation can be studied for perfumes but was not performed in this study. The mutagenicity response of the perfumes was in agreement with prior evidence of genotoxicants (ED_50_ of 1.3 μL perfume) using the SOS-Umu-C bioassay.^38^ The new HPTLC^fix^–duplex planar Ames–cytotoxicity bioassay–Vis method, using a 24-h incubation and more selective MTT substrate end point, was successful in detecting mutagens and cytotoxica in perfumes. By this, the previous method limitations (only a 5-h short incubation and an unselective bromocresol purple pH indicator end point) in the first attempt^45^ of a planar Ames format were overcome.

**Figure 8:**
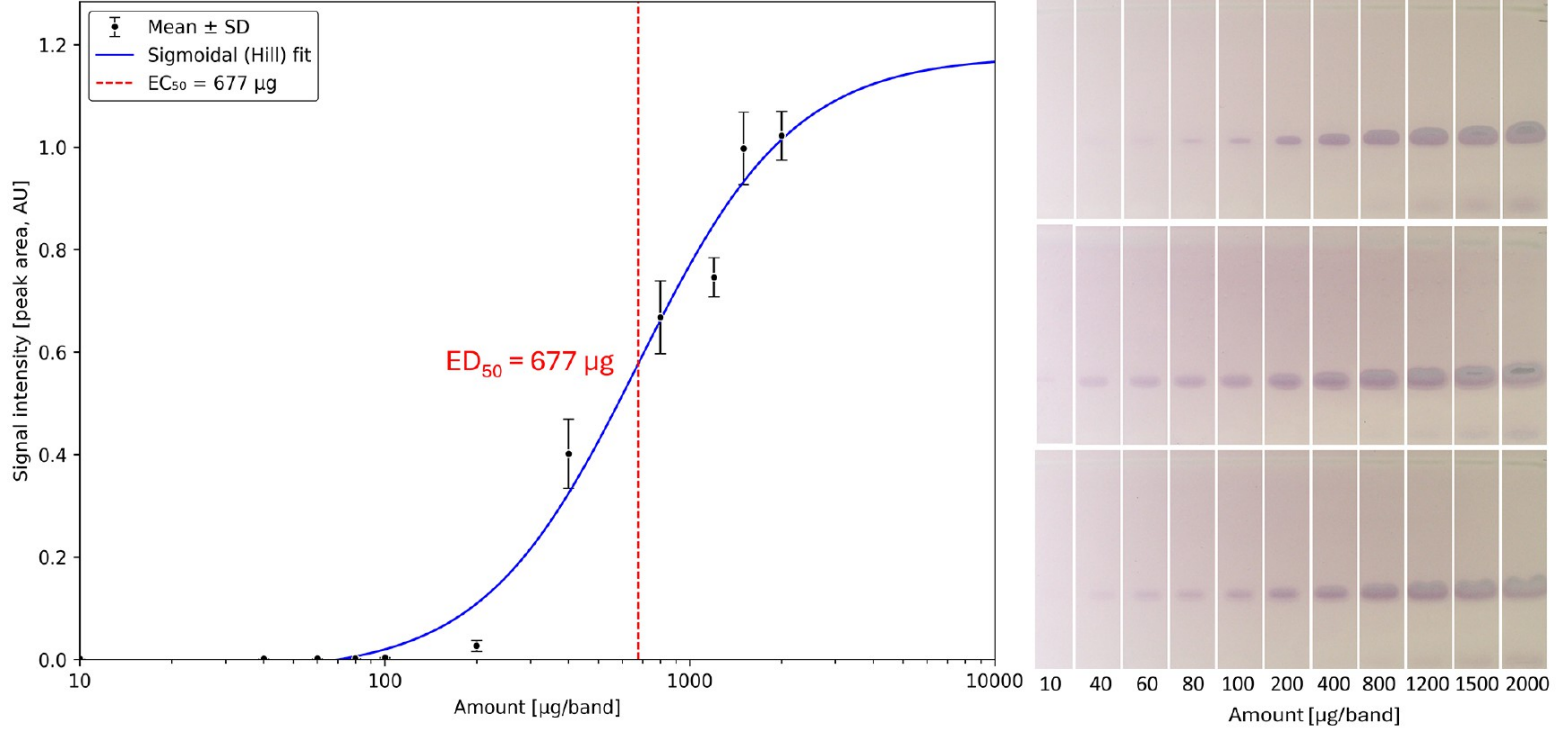
Quantitative dose–response study: mutagenicity bioautograms and the corresponding mean dose–response curve of skin care cream extract 1 (10–2000 μg/band, *n* = 3, separated as in Figure 4) via the HPTLC^fix^–duplex planar Ames–cytotoxicity bioassay–Vis using the *Salmonella* strain TA98, followed by videodensitometry (absorbance measurement of the three Vis chromatograms) to calculate the mean dose–response curve and ED_50_ to be 677 μg. Increasing associated cytotoxicity (zone whitening) was observed at amounts from 800–2000 μg/band.

### High-Throughput Screening

**F**or the first time, the new HPTLC–duplex planar S9–Ames–cytotoxicity bioassay–Vis workflow included testing for mutagenicity, cytotoxicity, and S9 metabolization (Figure 1). It enabled high-throughput screening of complex samples with minimal or no sample preparation, thereby indicating individual compounds and making product safety screening affordable. Screening complex samples has not been possible with the status quo technologies so far. The following scenario (Table 1) may illustrate this statement for a trained laboratory assistant in a routine setting. For status quo HPLC–HRMS–nanofractionation with associated bioassay testing of 22 complex samples for cytotoxicity and mutagenicity, a 30-min gradient separation of 22 complex samples takes 1 day (11 h). Each sample separation is subjected to the *in vitro* cytotoxicity assay via eluent splitter and nanofractionation. For 20-s fractions (133 μL given a 0.4 mL/min flow rate), 90 wells are filled per 30-min gradient for one sample, plus wells for negative and positive controls. Thus, for 22 samples, 22 96-well microtiter plates are required. Thus, the following *in vitro* cytotoxicity testing using the MTT substrate takes 2 days (2 dilutions, *i.e*., 2 × 96-well microtiter plates for each sample with 90 fractions). After dilution of the sample extracts to obtain cytotoxicity-free fractions, the time to result takes 7 days for the second *in vitro* assay, the Ames MPF mutagenicity testing (90 fractions/sample; 49.5 h for incubation, *i.e.*, 1.5 h preincubation and 48 h outgrowth phase).^10,11^ It requires 0.3 × 24-well microtiter plates and 1 × 384-well microtiter plates for a single sample fraction (for one assay setup with one strain and S9 metabolization; 6 dilutions prepared, Figure S8). Thus, 30 × 24-well microtiter plates and 90 × 384-well microtiter plates are consumed for 90 fractions of one sample, and for 22 samples, it takes 2,640 microtiter plates, which leads to vast plastic waste.

The analysis via ca. 120 microtiter plates for one sample/90 fractions (30 × 24-well and 90 × 384-well microtiter plates) sums up to 10 working days (2,5 days/2 × 96-well microtiter plates for cytotoxicity testing, plus 7,5 days/30 × 24-well/90 × 384-well microtiter plates for the Ames MPF assay testing, inclusive of the HPLC separation). The daily throughput is limited by the Ames MPF assay, given the parallel handling of 18 × 384-well microtiter plates per day, which takes 5 days for 90 fractions plus 48 h of incubation for the last incubation starting on day 5 (altogether 7 days), due to the required preincubation and transfer steps from 24-well to 384-well microtiter plates.

**Figure 9:**
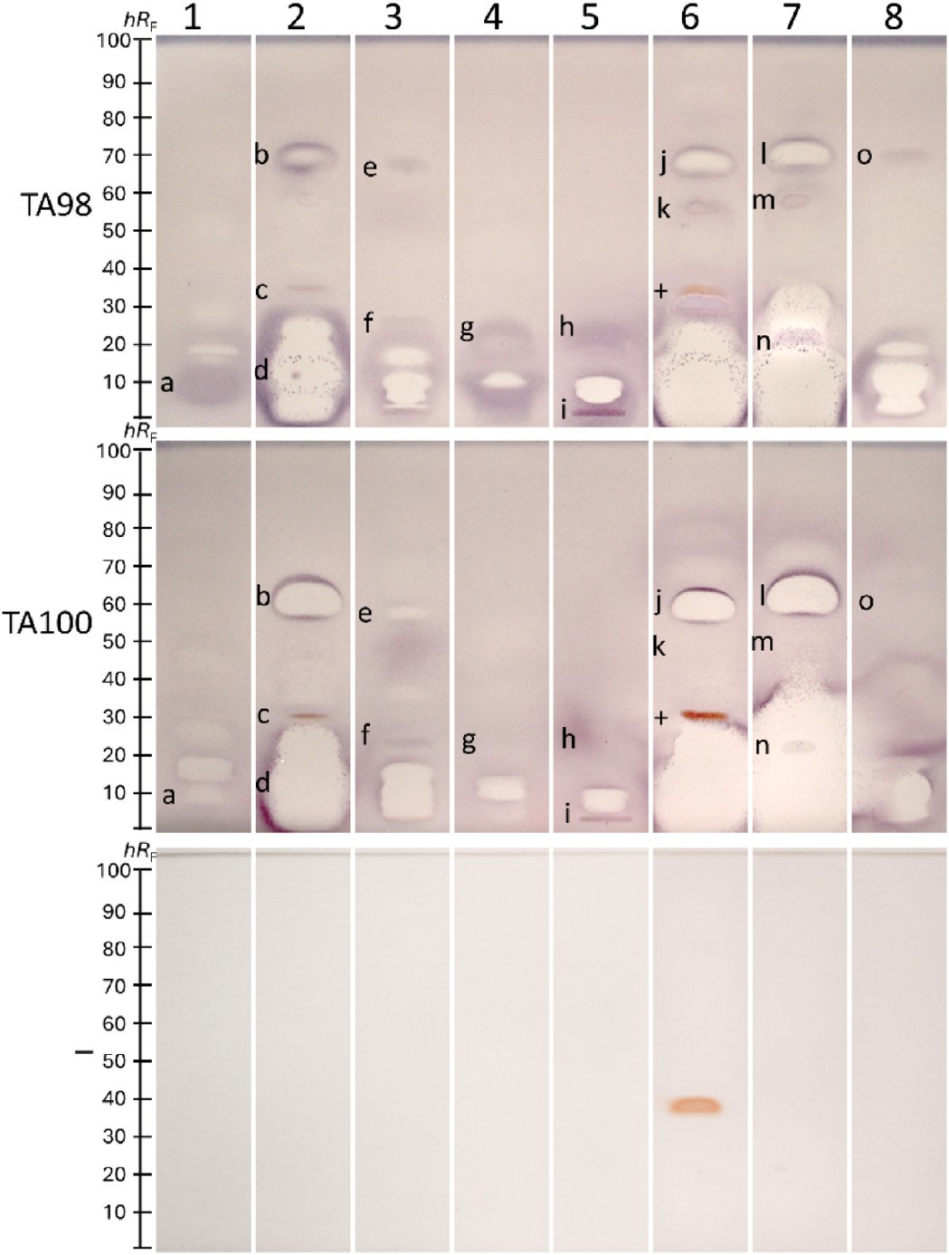
Mutagenicity/cytotoxicity bioautograms of the different perfumes 1–8 (Table S1, 1 μL/band each, developed on HPTLC silica gel 60 plates with cyclohexane–ethyl acetate 19:1, *V*/*V*) via the HPTLC^fix^–duplex planar Ames–cytotoxicity bioassay–Vis using *Salmonella* change strain TA98 and TA100, showing purple mutagen zones **a**–**o** and whitening yellowish cytotoxic compound zones. Verification of the bioassay response by the *Salmonella*-free negative control plate (false positives +) and positive controls (Figure S2).

In contrast, on one HPTLC plate, 22 complex samples were analyzed simultaneously, along with blanks and negative and positive controls, applied bandwise in the upper part of the plate (above the solvent front). The on-surface incubation of the sample compounds takes a total of 29 h, *i.e.*, 5 h with the strain, followed by 24 h together with the MTT to detect revertants (active metabolism). Thus, the incubation time (29 h) was reduced by 20 h compared to the Ames MPF *in vitro* assay (49.5 h), and was similar to the luminescent reporter strain *in vitro* assay.^12^ Highly advantageous was the simultaneous screening of 22 samples and duplex end point, making it a high-throughput screening for mutagenicity and cytotoxicity with minimal sample preparation of complex samples. The comparison of the time to result per sample (Figure 1, Table 1A) showed that the new duplex planar S9–Ames–cytotoxicity bioassay–Vis screening of 22 samples reduced 5-fold the total analysis time from 10 working days (Ames MPF and cytotoxicity *in vitro* bioassays) to 2 working days, whereof manual work was reduced 330-fold. The comparison of consumable costs per sample (Table 1B) showed 651-fold lower costs for the planar bioassay format, which worked almost plastic-free, supporting green analytical chemistry and sustainability goals. The costs and time savings are even higher for planar bioassay screening because absent or minimal sample preparation was not considered in the scenario. In contrast, sample preparation of complex samples is often a precondition for the column-based status quo (due to the reuse of the HPLC column) with associated *in vitro* assay testing, whereby the extended sample preparation is not only expensive, but also disadvantageous for the scientific outcome, as sample parts are lost.

For status quo analysis, matching an *in vitro* assay response with a respective HPLC-DAD-HRMS signal is complicated by different detection principles and sensitivities for sophisticated hyphenations.^83^ A wrong match can be generated, given the fact that an active compound is not necessarily absorbing and/or ionizable, or a highly active trace-level compound can easily be hidden and overlooked in the HRMS background signals (the ionizability of compounds can differ by 10 million, and ionizability is not equivalent to activity). Nevertheless, after about a month (in total), the status quo analysis would provide an equivalent outcome as obtained by one planar bioautogram, the latter with a comparatively much easier readout (effect first) and fully automated transfer of the active substance zone to HRMS, which is highly targeted and efficient. The duplex planar screening detects mutagens and cytotoxica concurrently. It does not need a dose-related response increase to be certain of an effect response due to the integrated separation from matrix interferences.

**Table 1:** Comparison of the Time to Result per Sample (A) and Consumable Costs per Sample (B, Market Prices in 2025) via HPLC–MS–Nano–Fractionation Plus Associated *In Vitro* Bioassay *versus* HPTLC–Duplex Planar Ames–Cytotoxicity Bioassay–Vis; Scenario Calculated for a Trained Laboratory Assistant in a Routine Setting, Excluding the Overnight Cultivation, Which is the Same for Both

**A**	**Time to result per sample**	**HPLC–MS–nano- fractionation + Ames MPF bioassay** **(90 fractions/sample)**	**HPLC–MS–nano- fractionation + cytotoxicity bioassay** **(90 fractions/sample)**	**HPTLC–duplex planar Ames–cytotoxicity bioassay**
	Sample preparation	Required	Required	None or minimal
	Separation	0.5 h	0.5 h	1.5 h, but for 22 samples
	Bioassay	7 days^a^	2 days^a^	29 h, but for 22 samples
	Total	7.5 days	2.5 days	2 days, but for 22 samples
	**Factor saved**	-	-	**5-fold less**
	Thereof manual operations	4.5 days	1 days	0.15 h (3.3 h/22 samples)
	**Factor saved**	-	-	**330-fold less**
**B**	**Consumables costs (€) per sample**			
	Stationary phase	3.0^b^	3.0^b^	1.0^c^
	Solvents	5.0^d^	5.0^d^	0.1^e^
	96-Well plate	2.5^f^	-	-
	24/384 + 96-Well plates	882.0^g^	5.0^h^	-
	S9 metabolization	120.0^i^	-	0.6^j^
	Medium	27.0^k^	2.0^l^	0.2^m^
	Substrate	180.0^n^	2.0^o^	<0.0
	Total per sample	1219.5	17.0	1.9
	**Factor saved**	-	-	**651-fold less**

a Pipetting 2 × 96-well microtiter plates, followed by the cytotoxicity assay, took 2 days; pipetting 30 × 24-well and 90 × 384-well microtiter plates, followed by the Ames MPF assay, took 7 days (for simplification, only one strain is used, with rat liver S9 metabolization, including a 49.5 h incubation); the cost of pipette material is negligible.

b The C18 column cost 1200 €, with lifetime 400 runs for highly complex samples.

c For the duplex bioassay (for simplification, only one strain plate and one negative control plate), 2 HPTLC plates were required (11.0 €/plate divided by 22 samples results in 0.5 € × 2 HPTLC plates).

d 30 min gradient, 2 mL/min (mixed calculation 83 €/L).

e On average 10 mL of solvents (mixed calculation 83 €/L) per HPTLC plate, divided by 22 samples × 2 HPTLC plates.

f One 96-well microtiter plate (2.5 € each) is required to collect 90 fractions; the resulting volume is sufficient to perform the cytotoxicity and Ames MPF bioassay.

g 90 factions mean 30 × 24-well microtiter plates (3 fractions each with 6 dilutions) and 90 × 384-well microtiter plates (with one fraction each), summing up to 120 microtiter plates (for 24-well plates, 2.4 € each × 30 + for 384-well plates, 9 € each × 90).

h 90 fractions also mean 2 × 96-well microtiter plates (2 dilutions, with 2.5 € × 2 each).

i 4.0 € for 135 μL of rat liver S9 per 24-well microtiter plate × 30.

j 6.6 € for 750 μL of rat liver S9 per HPTLC plate × 2 divided by 22 samples.

k 0.3 € for 17.5 mL of deficiency medium per microtiter plate × 90 microtiter plates.

l 1.0 € for 17.5 mL of nutrient broth per microtiter plate × 2 microtiter plates.

m 2.5 mL per HPTLC plate × 2 divided by 22 samples.

n 2.0 € for 50 mg/mL bromocresol purple per microtiter plate × 90 microtiter plates.

o 1.0 € for 2 mg/mL MTT per microtiter plate × 2 microtiter plates.

### Method Transfer to the Sustainable 2LabsToGo-Eco

To improve sustainability, the duplex planar Ames-cytotoxicity bioassay–Vis method, including HiCultS9 metabolization, was transferred to the affordable all-in-one open-source 2LabsToGo-Eco, which is the most sustainable lab of the future.^51^ The mobile phase cyclohexane–methyl-*tert*-butyl ether 4:1, *V*/*V,* and instrumental settings were used from a previous 2LabsToGo-Eco method.^41,51^ The six cosmetic and skin care cream products 5 and 17–21 selected had not previously been tested with HiCultS9 metabolization using conventional HPTLC instrumentation and thus were analyzed by both systems for comparison (Figure 10). It was not expected to obtain the same bioautogram profiles with regard to the *hR*_F_ value and zone intensity due to the differences, *i.e*., not the exact same application amounts, different mobile phases, and thus separation selectivity, and reduced vapor space in the horizontal development *versus* conventional vertical development. Nevertheless, the same active zones **a**–**g** were consistently detected in both systems, although with more or less intensity. The mutagenicity and cytotoxicity profiles of the cosmetics and skin care cream products were reliably assessed, confirming the suitability for rapid and sustainable screening using a low-cost and portable 2LabsToGo-Eco, while reducing cost, material, and infrastructure requirements. It illustrated that high-quality safety testing is feasible outside of high-end laboratory infrastructure, which offers a clear perspective for future analyses in a broad area.

**Figure 10:**
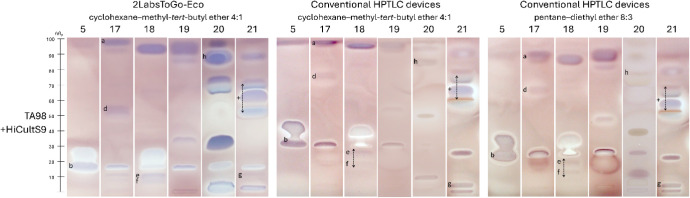
2LabsToGo-Eco mutagenicity/cytotoxicity bioautogram of 6 different cosmetic and skin care cream products (Table S1, 0.8 mg band each, separated on HPTLC plate silica gel 60 with cyclohexane–methyl-*tert*-butyl ether 4:1, *V*/*V*) reveals mutagen zones **a**–**h** by the duplex planar Ames–cytotoxicity bioassay–Vis using the *Salmonella* strain TA98 and HiCultS9 metabolization.

### Progress Achieved

Complex or lipophilic samples, such as personal care products including oils, balms, and wax-based formulations, are difficult to assess due to limited solubility in aqueous-buffered and salt-rich media of conventional *in vitro* assay methods. As a result, some of the sample components may not be accessible to the test organisms. Hazardous effects observed for hardly dissolvable sample parts that are prone to solidification are highly underrated. The drawbacks of *in vitro* assay sum values (impaired by insolubility, solidification, precipitation, micelle formation, adsorption to plastics, associated cytotoxicity, false positives, false negatives, *etc*.) have already been discussed.^1^ Furthermore, HPLC–HRMS/MS methods are widely used for substance identification and quantification^84^ but only detect what is chemically ionizable and detectable. The response depends on the ionization potential of a chemical structure, and the detected signal is not necessarily the signal that is present and causes the mutagenic effect. Non-ionizable compounds, highly active trace-level compounds, or active substances outside the target scope are often overlooked and remain undetected if only concentration-/ionization-based analytical methods are applied, as recently discussed.^1^ Effect-based screening methods have been neglected in product safety assessment for a long time due to such method limitations for complex samples and the high costs involved. The planar bioassays address this gap. On the same adsorbent surface, they allow not only the chromatographic separation of complex mixtures but also the detection of zone-specific effects. Lipophilic compounds with *n*-octanol/water partition coefficients of >10 can be analyzed. Effect-directed detection at an early stage in the workflow is crucial. It points, independent of the ionization potential of a substance, to highly active compounds, even at the trace level.^1^ The approach is suitable for routine screening, quantification, and advanced structural analysis to unmask hazardous substances that remain otherwise hidden by conventional analytical techniques.

## Conclusions

For the first time, a rapid, sensitive, selective, and quantitative mutagenicity and cytotoxicity screening of complex samples was enabled by the new HPTLC–duplex planar Ames–cytotoxicity bioassay–Vis method, including S9 metabolization. Mutagens and cytotoxica were simultaneously revealed in three quite different, highly complex product groups, which successfully proved broad applicability. Mutagens and cytotoxica were already detected for 3 mg of polyphenol-rich tea, 0.8 mg of lipophilic cosmetics or skin care cream, and 1 μL of volatile perfume, which is alarming considering the larger quantities consumed or applied daily. A possible entry path into the body can be hair follicles, skin irritations, and microinjuries caused by shaving body hair, which often necessitate the application of skin care products, thereby exceeding the ED_50_ for mutagenicity by 4 orders of magnitude. However, such a simplified comparison is not a quantitative risk assessment. Unfortunately, no substantial detoxification by simulated metabolization via the human liver S9 enzyme was revealed. Using the affordable and sustainable open-source 2LabsToGo-Eco helps to start with the new planar bioassay technology as a valuable tool in regulatory safety and risk assessments (*e.g*., screening of chemicals, food, feed, lifestyle products, personal care products, and food contact materials), industrial quality control (*e.g*., checking of raw materials and final products for hazardous impurities or contaminants), drug development (*e.g*., early-stage mutagenicity screening), and environmental screening (*e.g*., mutagenicity screening of industrial wastewater effluents into surface water). Since five plates had to be performed (for TA98 and TA100, both with S9, and a negative control plate), future developments will focus on an all-on-the-same-plate solution, further increasing the throughput of this already highly efficient duplex planar Ames–cytotoxicity bioassay.

## Supplementary Material



## Data Availability

Data will be made available upon request.

## Notes

Detailed list of tested samples (Table S1); data on method optimization (Figure S1), positive controls (Figure S2), genotoxicity bioautogram of tea samples (Figure S3), and references (Figure S7); dose–response curves of mutagenicity/cytotoxicity bioautograms (Figure S4); Vis chromatogram for comparison (Figure S5); mutagenicity/cytotoxicity bioautogram before and after derivatization of cosmetic and skin care cream extracts (Figure S6); and status quo testing for comparison with the new method in Table 1 (Figure S8) (PDF)

## Notes

Dedicated to the life work of Prof. Dr. Colin Poole, Wayne State University, Detroit, USA.
