# Supplementary material for: High-Throughput Testing for Unknown Mutagens and Cytotoxica via Duplex Planar Ames–Cytotoxicity Bioassay Including Metabolic S9 Activation
Source: Anal Chem. 2026 Mar 2;98(10):7374–91. doi: 10.1021/acs.analchem.5c06690 (PMC13000881; doi:10.1021/acs.analchem.5c06690)
Supplement: Supplementary file 1 [file ac5c06690_si_001.pdf]

Supporting Information

High-Throughput Testing for Unknown Mutagens and Cytotoxica via Duplex Planar Ames-Cytotoxicity Bioassay Including Metabolic S9 Activation

Katharina Schmidtmann[a], Ann-Cathrin Kayser[a], Gertrud E. Morlock[a,]*

[a]Chair of Food Science, Institute of Nutritional Science, Justus Liebig University Giessen, Heinrich-Buff-Ring 26-32, 35392 Giessen, Germany

Dedicated to the lifework of Prof. Dr. Colin Poole, Wayne State University, Detroit, USA

*Corresponding author. Tel.: +49 641 9939140, fax: +49 641 9939149, E-mail address: gertrud.morlock@uni-giessen.de (G.E. Morlock)

Table of Contents

Table S1	List of tested cosmetic and skin care creams, perfumes, and teas	S3
Figure S1	Study of different second incubation times and different MTT volumes	S4
Figure S2	Analysis of positive controls applied as bands	S5
Figure S3	HPTLC–planar SOS-Umu-C–Vis/FLD 254 nm bioautograms of tea samples	S6
Figure S4	Dose-response study: mutagenicity/cytotoxicity bioautograms at Vis of skin care cream	S7
Figure S5	Chromatogram at Vis of cosmetic and skin care cream extracts	S8
Figure S6	Duplex planar Ames-cytotoxicity bioautogram at Vis and chromatogram at FLD 366 nm of the subsequently applied primuline reagent	S9
Figure S7	HPTLC–planar SOS-Umu-C–FLD 254 nm bioautogram of references	S10
Figure S8	Status quo testing for comparison with the new method in Table 1	S11

Table S1. List of tested cosmetic and skin care creams 1–22, perfumes 1–8, and teas 1–5; product names and manufactures were not published to avoid damage to companies.

ID	Cosmetic and skin-care creams	Perfumes for women	Teas
1	Face cream	Eau de parfum<br>Top notes: pepper, bergamot<br>Middle notes: patchouli, angelica, coumarin<br>Base notes: musk, ambergris	Earl grey tea
2	Face cream	Eau de parfum<br>Top notes: pink pepper<br>Middle notes: freesia, peony<br>Base notes: animal, musk	Green tea
3	Face cream	Eau de parfum<br>Top notes: lavender, rose, geranium, lily orange blossom, rose, jasmine<br>Middle notes: clove, vetiver, laurel<br>Base notes: Virginia cedar, musk, sandalwood	Rooibos tea
4	Body butter	Eau de Cologne<br>pink pepper, grapefruit	Herbal tea
5	Hand cream	Eau de toilette<br>Top notes: ylang-ylang<br>Middle notes: lily of the valley<br>Base notes: jasmine, musk	Herbal tea organic
6	Hand cream	Eau de toilette<br>Top notes: Tanzanian ginger, bitter orange<br>Middle notes: peony, damask rose, jasmine sambac<br>Base notes: vanilla, Venezuelan tonka bean	
7	Face cream	Eau de parfum<br>Top notes: lemon, bergamot<br>Middle notes: lemon, jasmine<br>Base notes: jasmine, cedar	
8	Face cream	Eau de parfum<br>Top notes: cactus blossom<br>Middle notes: rose buds<br>Base notes: cedarwood	
9	Face cream		
10	Make up		
11	Mascara		
12	Lipstick		
13	Lip gloss		
14	Lipstick		
15	Make up		
16	Face cream		
17	Medical ski cream		
18	Hand cream		
19	After sun lotion		
20	Tanning cream		
21	Sun cream		
22	Sun cream		

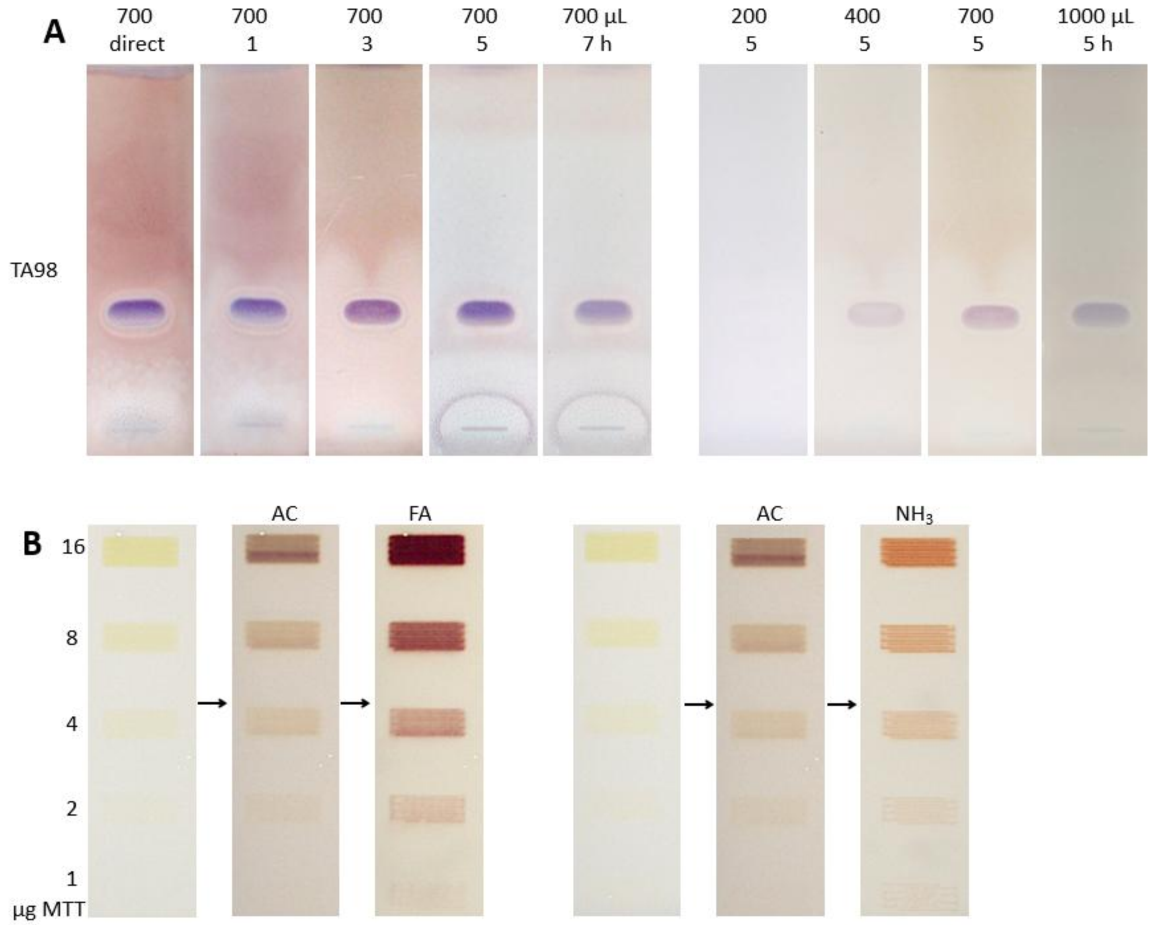

Figure S1. Study of different second incubation times (1–7 h) and different MTT volumes (200–1000 µL): mutagenicity/cytotoxicity bioautograms of the face cream 1 (Table S1, 0.8 mg/band each, separated as in Fig. 4) via the HPTLC^{fix}–duplex planar Ames-cytotoxicity bioassay–Vis using the *Salmonella* strain TA98 and 24-h second incubation time, showing a prominent purple mutagenic substance zone and whitening yellowish cytotoxic compound zone; a second incubation time of 5 h (to reach a bright plate background) and a 700 µL MTT volume were selected (**A**). Applied amounts of MTT (1–16 µg) on two HPTLC silica gel 60 plates were reduced with 100 mM ascorbic acid solution (AC) to form the formazan, which was then exposed either to vapor of 400 µL formic acid (FA, 20 min, plate pH 3), resulting in a strong purple coloration, or to vapor of 400 µL ammonia (NH_3, 25%, 20 min, plate pH 9), resulting in a reddish-orange coloration (**B**).

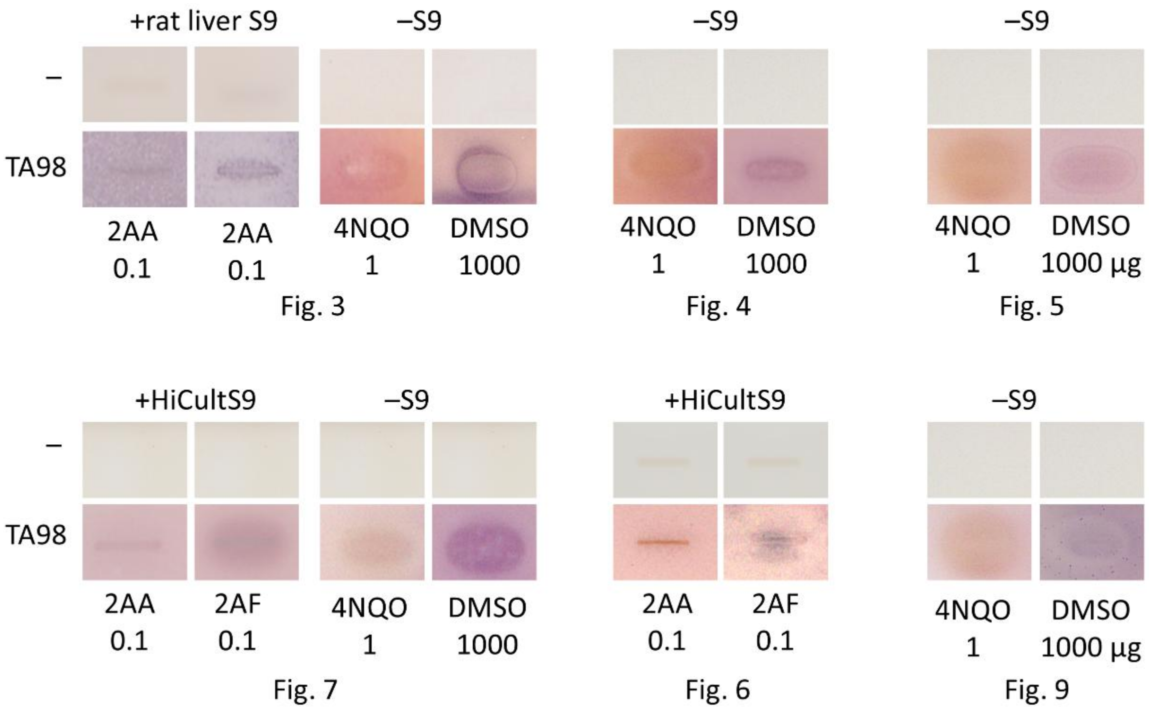

Figure S2. Analysis of positive controls applied as bands on each upper plate part (cut off in Figs. 3–9) for proof of the proper performance of the duplex planar Ames-cytotoxicity bioassay–Vis via the *Salmonella* Typhimurium strain TA98 without (-S9; 4NQO 1 µg/band and DMSO 1000 µg/band) and with metabolization (2AA and 2AF 0.1 µg/band each) using the rat liver enzyme S9 or HiCultS9 systems.

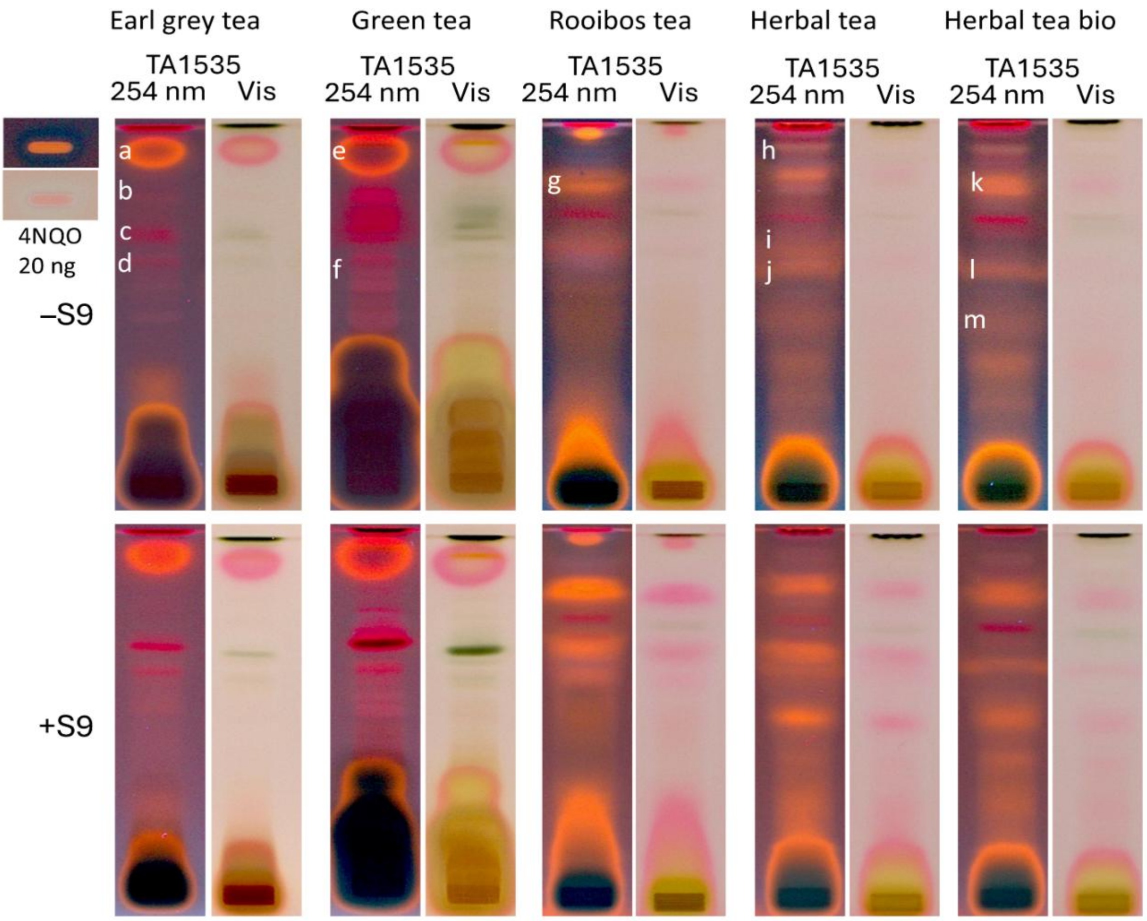

Figure S3. HPTLC–planar SOS-Umu-C–Vis/FLD 254 nm bioautograms via *Salmonella* strain TA1535 of tea extracts 1–5 (Table S1, 3 mg/band) without (-S9) and with metabolization (+S9) using the rat liver enzyme system, developed on HPTLC plate silica gel 60 with dichloromethane–methanol–ammonia, 85:15:1, *V*/*V*/*V*, showing genotoxic orange fluorescent zones at FLD 254 nm and rose zones at Vis; the proper bioassay performance was verified by the positive control 4NQO (20 ng/band).

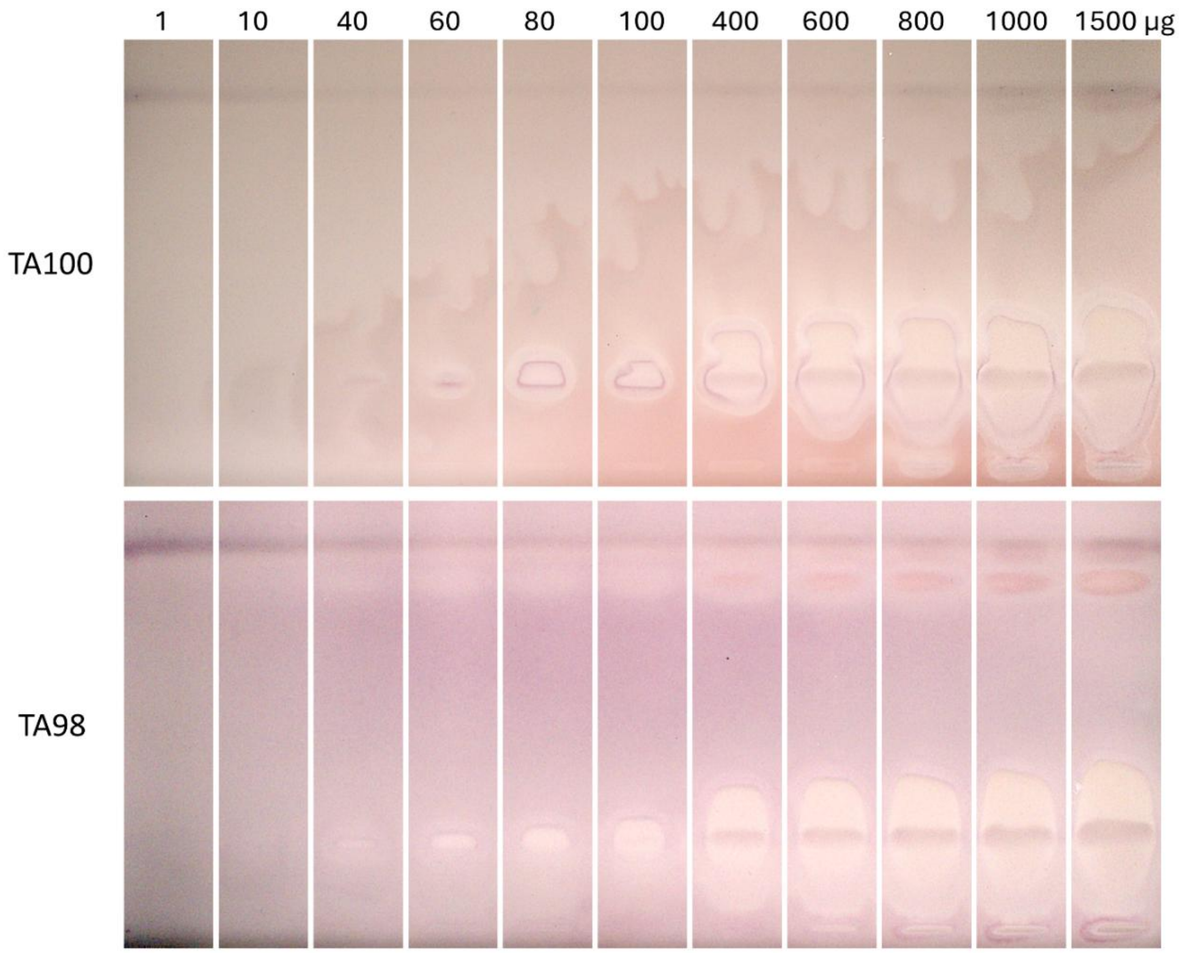

Figure S4. Dose-response study: mutagenicity/cytotoxicity bioautograms at Vis of skin care cream extract 5 (increasing 1–1500 µg/band, separated as in Fig. 4) by the HPTLC^{fix}–duplex planar Ames-cytotoxicity bioassay–Vis via *Salmonella* strains TA98 or TA100.

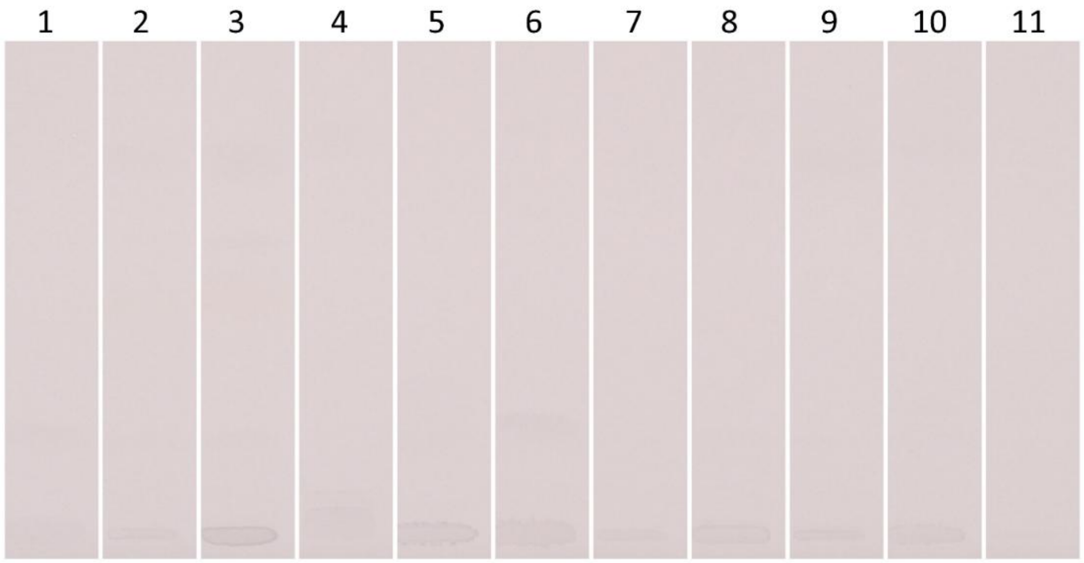

Figure S5. Chromatogram at Vis of cosmetic and skin care cream extracts 1–11 (Table S1, 0.8 mg/band each, separated as in Fig. 4) showing almost no compound zones.

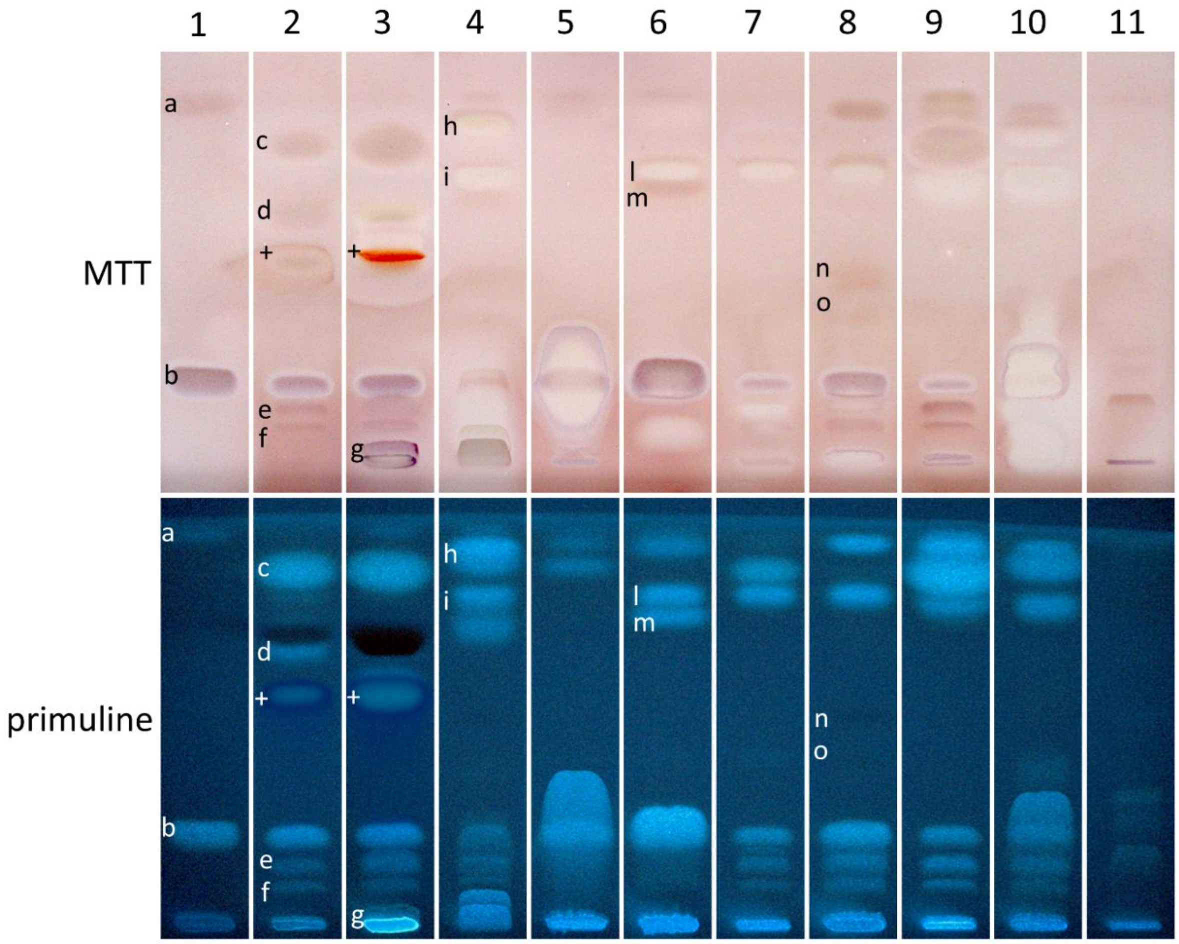

Figure S6. HPTLC[fix]–duplex planar Ames-cytotoxicity–Vis bioautogram via *Salmonella* strain TA98, and after additional derivatization with the primuline reagent on the same bioautogram, chromatogram at FLD 366 nm: cosmetic and skin care cream extracts 1–11 (Table S1, 0.8 mg/band each, separated as in Fig. 4) show purple mutagen zones **a**–**o** and whitening yellowish cytotoxic compound zones; on the same bioautogram, the primuline reagent detected lipophilic compound zones **a**–**m** at FLD 366 nm.

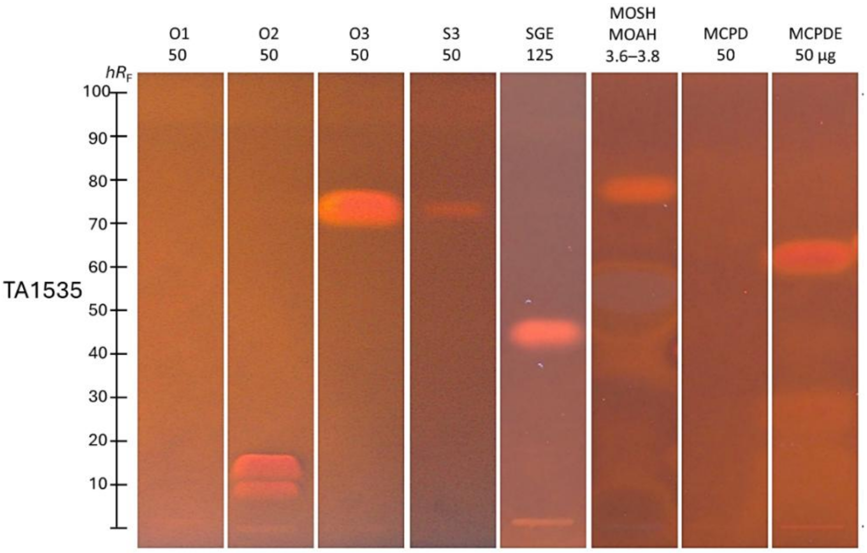

Figure S7. HPTLC–planar SOS-Umu-C–FLD 254 nm bioautogram via *Salmonella* strain TA1535 of reference standards (MAG candidate O_1, DAG candidate O_2, TAG candidates O_3 and S_3, all 50 µg each; SGE, 125 µg; MOSH/MOAH mixture, 3.6–3.8 µg; MCPD and MCPDE, 50 µg each), developed on an HPTLC silica gel 60 plate with pentane – diethyl ether, 8:3, *V*/*V,* showing orange fluorescent genotoxic compound zones.

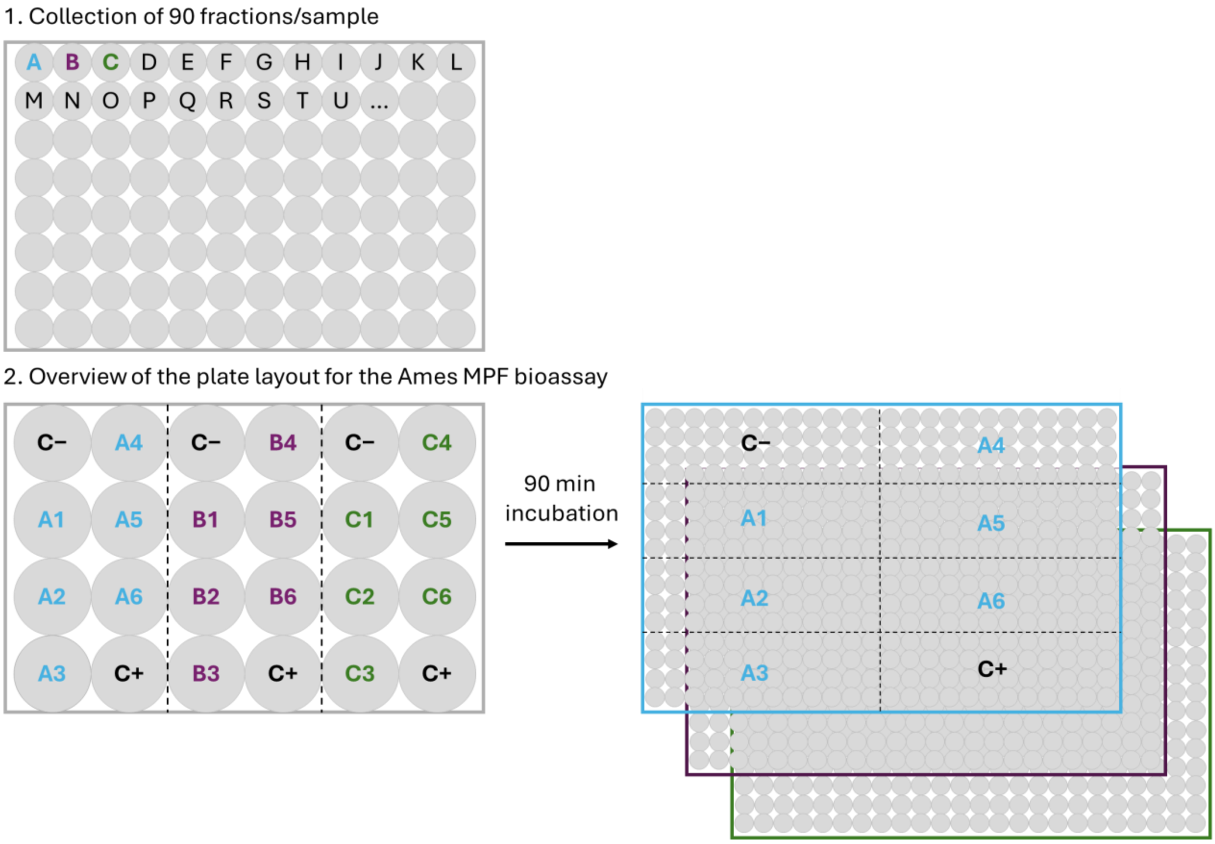

Figure S8. Status quo testing for comparison with the new method in Table 1: **(1)** Representative plate layout illustrating the collection of 90 fractions in a 96-well microtiter plate; fractions A–U are shown exemplarily; fraction numbering continues consecutively up to 90, as a total of 90 fractions are collected per sample. **(2)** The plate layout scheme for the Ames MPF assay; three fractions (A–C) are initially distributed into a 24-well microtiter plate, each tested at six dilution levels (A1–A6, B1–B6, C1–C6), together with positive (C+) and negative (C–) controls. After 90 min of incubation, the six dilution wells of each fraction, as well as the corresponding positive and negative controls, are transferred into a 384-well microtiter plate, followed by a 48-h incubation and endpoint readout. Accordingly, one fraction is analyzed per 0.3 x 24-well microtiter plate and 1 x 384-well microtiter plate.
